# Supplementary material for: Studying the Long-term Impact of COVID-19 in Kids (SLICK). Healthcare use and costs in children and young people following community-acquired SARS-CoV-2 infection: protocol for an observational study using linked primary and secondary routinely collected healthcare data from England, Scotland and Wales
Source: BMJ Open. 2022 Nov 10;12(11):e063271. doi: 10.1136/bmjopen-2022-063271 (PMC9659708; doi:10.1136/bmjopen-2022-063271)
Supplement: Supplementary data [file bmjopen-2022-063271supp001.pdf]

Studying the Long-term Impact of COVID-19 in Kids (SLICK). Healthcare use and costs in children and young people following community-acquired SARS-CoV-2 infection: protocol for an observational study using linked primary and secondary routinely collected healthcare data from England, Scotland and Wales.

Supplementary Information

Supplementary Table 1 – Datasets available and Trusted Research Environments

Country	Trusted Research Environment	Datasets and linkages
Scotland	Scottish National Safe Haven	SMR00 – Outpatient appointments and attendances<br>SMR01 – General acute inpatient and day case<br>SMR04 – Mental health inpatient and day case<br>SMR06 – Scottish cancer registry<br>COVID Tests – Laboratory SARS-CoV-2 tests<br>Prescribing Information System – Community prescriptions<br>Accident and Emergency Datamart<br>GP out of hours<br>Scottish Ambulance Service<br>NHS24 calls<br>NRS Deaths<br>NRS Infant deaths<br>EAVE II – Scheduled and unscheduled primary care<br>ISARIC4C/CO-CIN<br>COGUK – SARS-CoV-2 variant<br>TVMT – SARS-Cov-2 vaccination data
England	OpenSAFELY	TPP - Primary Care<br>SGSS COVID testing data<br>ONS death certificates – available from 2019-02-01<br>SUS APCS (inpatient hospital) – available from 2016-04-01<br>SUS OPA (outpatient hospital) – available from 2019-04-01<br>SUS ECDS (emergency care) – available from 2017-10-01<br>ISARIC4C/CO-CIN
Wales	SAIL	ConCOV - Wales Multimorbidity Cohort (WMC) - COVID-19<br>WLGP – Primary care<br>PEDW – Secondary care (inpatient & day case)<br>ADDE – ONS mortality data<br>CCDS – Critical care<br>CDDS – Consolidate deaths from COVID-19

		**CENW** – Census 2011<br>**CTTP** – COVID-19 test, trace and protect<br>**CVLF** – COVID-19 lateral flow tests<br>**CVSP** – COVID-19 shielded people<br>**CVVD** – COVID-19 vaccines<br>**EDDD** – Emergency department (daily)<br>**EDDS** – Emergency department<br>**ICCD** – intensive care national audit (COVID only admissions)<br>**ICNC** – intensive care national audit<br>**MIDS** – Maternity initial screening and birth<br>**NCCH** – National community child health (maternity, childbirth, etc)<br>**NHSO** – NHS 111, out of hours<br>**OPDW** – Outpatients<br>**OPRD** – Outpatient referrals<br>**PATD** – COVID-19 lab tests<br>**RTTD** – Referral to treatment times<br>**WASD** – Welsh ambulance service<br>**WCSU** – Welsh cancer incidence surveillance unit<br>**WDDS** – Welsh prescription dispensing<br>**WDSD** – Individuals registered with GP, addresses/household information<br>**CRCS** – Children in care or receiving support register<br>**CYFI** – Cystic Fibrosis register<br>**BREC** – Register of all children in Wales with type 1 diabetes<br>**ISARIC4C/CO-CIN**

***Abbreviations: SMR**: Scottish Morbidity Record, **NRS**: National Records of Scotland **EAVE-II:** Early Pandemic Evaluation and Enhanced Surveillance of COVID-19, **ISARIC/CO-CIN**: International Severe Acute Respiratory and emerging Infection Consortium / COVID-19 Clinical Information Network, **COGUK:** COVID-19 Genomics UK Consortium, **TMVT**:Turas Vaccination Management Tool, **NHS**: National Health Service, **TPP:** The Phoenix Partnership (GP group), **SGSS**: Second Generation Surveillance System, **ONS**: Office for National Statistics, **SUS**: Secondary Use Services, **APCS**: Admitted patient care statistics, **OPA**: Outpatient attendances, **ECDS**: Emergency care datasets, **SAIL:** Secure Anonymised Information Linkage, **WLGP**: Welsh Longitudinal General Practice, **PEDW**: Patient Episode Database for Wales, **ADDE**: Annual District Death Extract, **CCDS:** Critical Care Data Source, **CDDS:** COVID-19 Consolidated Deaths, **CENW:** Office of National Statistics Census, **CTTP:** COVID-19 Test, Trace & Protect, **CVLF:** COVID-19 Lateral Flow, **CVSP:** COVID-19 Shielded People, **CVVD:** COVID-19 Vaccine Data, **EDDD:** Emergency Department Dataset Daily, **EDDS:** Emergency Department Dataset, **ICCD:** Intensive Care National Audit & Research Centre (ICNARC) - COVID only admissions, **ICNC:** Intensive Care National Audit & Research Centre (ICNARC), **MIDS:** Maternity Indicators Dataset, **NCCH:** National Community Child Health, **NHSO:** NHS 111 Call data, **OPDW:** Outpatient Dataset for Wales, **OPRD:** Outpatient Referral Dataset, **PATD:** Pathology Data (COVID-19 daily), **RTTD:** Referral to Treatment Times Dataset, **WASD:** Welsh Ambulance Service Dataset, **WCSU:** Welsh Cancer Incidence Surveillance Unit, **WDDS:** Welsh Dispensing Dataset, **WDSD:** Welsh Demographic Service Dataset.*

Supplementary Figure A – Dataflow diagram for Wales

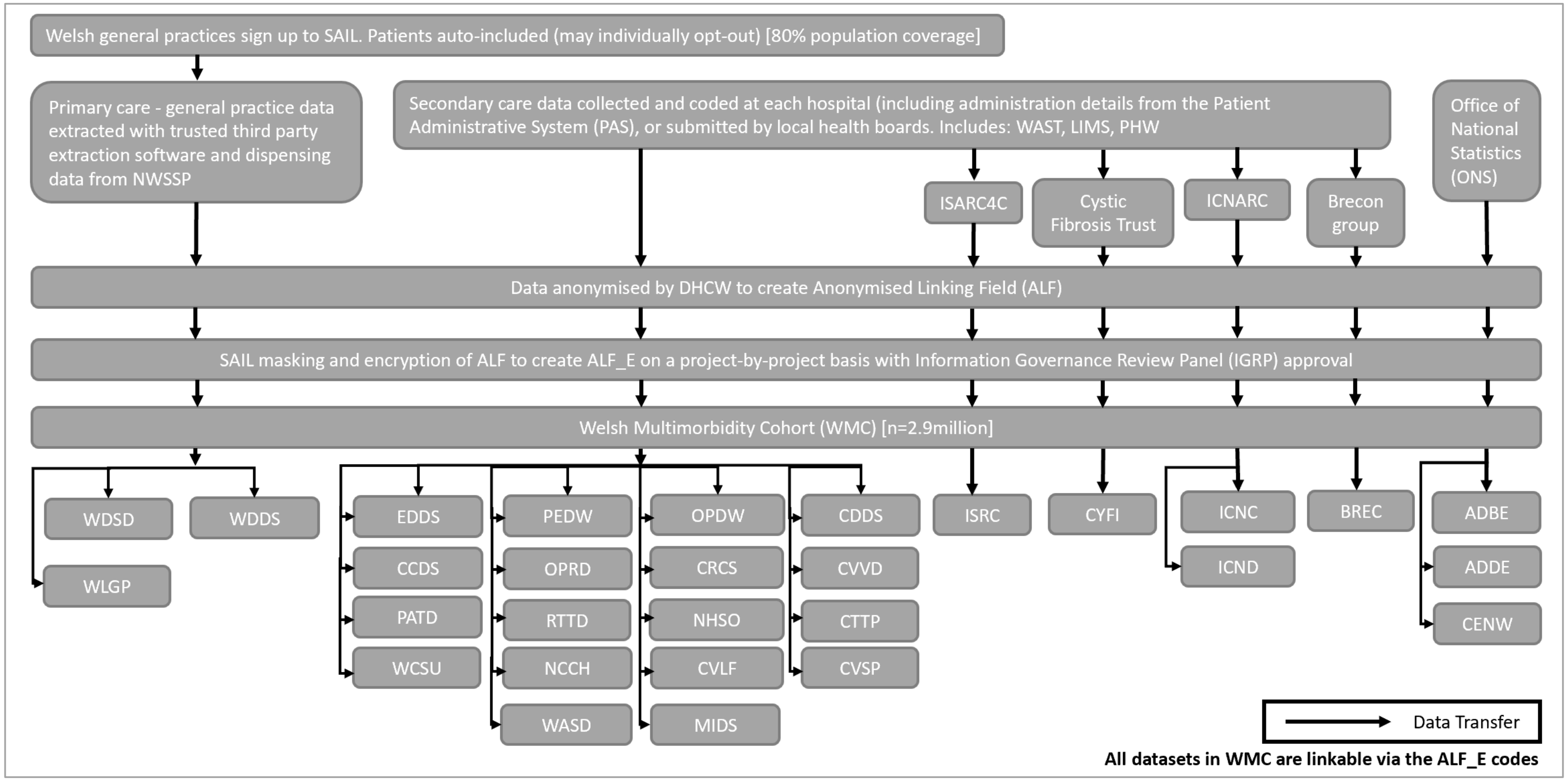

Abbreviations: SAIL: *Secure Anonymised Information Linkage,* ***WLGP****: Welsh Longitudinal General Practice,* ***PEDW****: Patient Episode Database for Wales,* ***ADDE****: Annual District Death Extract,* ***BREC:*** *Brecon Cohort (Children with type 1 diabetes register),* ***CCDS:*** *Critical Care Data Source,* ***CDDS:*** *COVID-19 Consolidated Deaths,* ***CENW:*** *Office of National Statistics Census,* ***CRCS:*** *Children Receiving Care & Support Services,* ***CTTP:*** *COVID-19 Test, Trace & Protect,* ***CVLF:*** *COVID-19 Lateral Flow,* ***CVSP:*** *COVID-19 Shielded People,* ***CVVD:*** *COVID-19 Vaccine Data,* ***CYFI:*** *Cystic Fibrosis Register,* ***EDDS:*** *Emergency Department Dataset,* ***ICCD:*** *Intensive Care National Audit & Research Centre (ICNARC) - COVID only admissions,* ***ICNC:*** *Intensive Care National Audit & Research Centre (ICNARC),* ***ISRC:*** *International Severe Acute Respiratory & Emerging Infection Consortium,* ***ISARIC4C:*** *International Severe Acute Respiratory & Emerging Infection Consortium (Coronavirus Clinical Characterisation Consortium),* ***MIDS:*** *Maternity Indicators Dataset,* ***NCCH:*** *National Community Child Health,* ***NHSO:*** *NHS 111 Call data,* ***OPDW:*** *Outpatient Dataset for Wales,* ***OPRD:*** *Outpatient Referral Dataset,* ***PATD:*** *Pathology Data (COVID-19 daily),* ***RTTD:*** *Referral to Treatment Times Dataset,* ***WASD:*** *Welsh Ambulance Service Dataset,* ***WCSU:*** *Welsh Cancer Incidence Surveillance Unit,* ***WDDS:*** *Welsh Dispensing Dataset,* ***WDSD:*** *Welsh Demographic Service Dataset.*

Supplementary Figure B – Dataflow diagram for Scotland

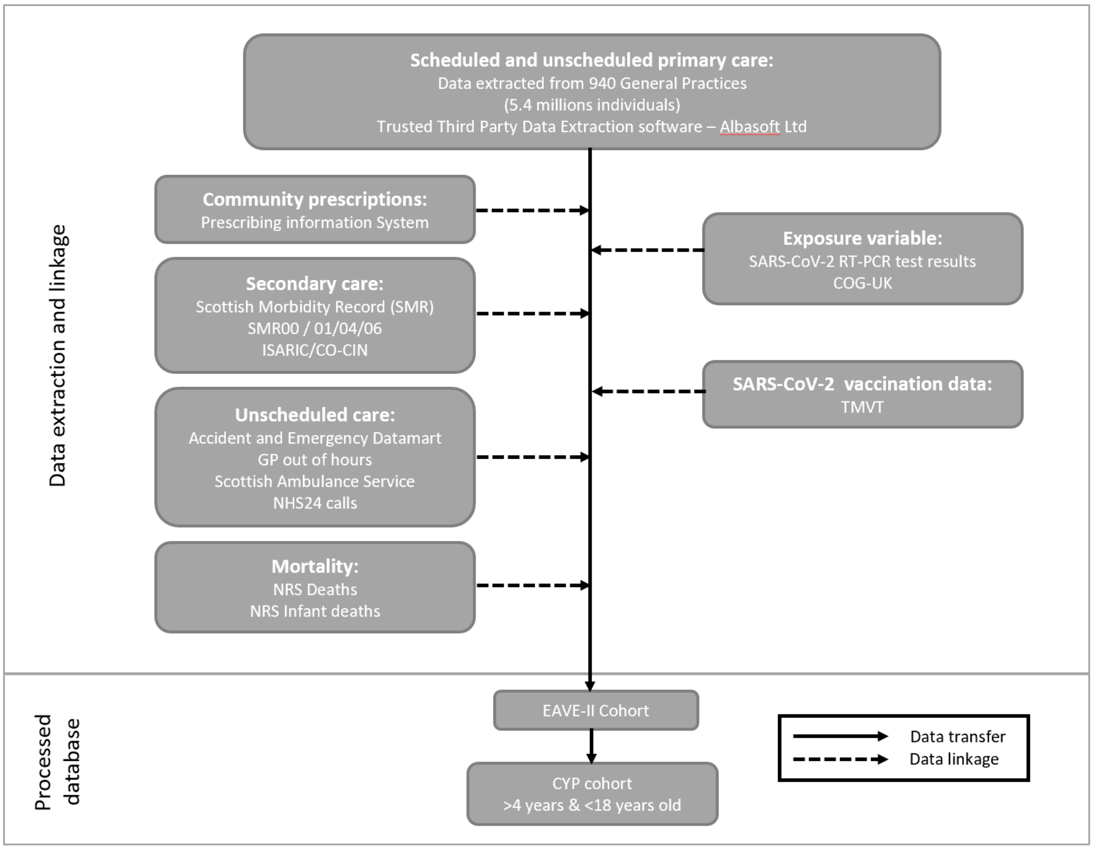

***Abbreviations: ISARIC/CO-CIN**: International Severe Acute Respiratory and emerging Infection Consortium / COVID-19 Clinical Information Network, **GP**: general practice; **NHS**: National Health Service, **NRS**: National Records of Scotland; **RT-PCR**: reverse transcription polymerase chain reaction, **TMVT**: Turas Vaccination Management Tool; **COG-UK:** Centre of Genomics United Kingdom, **CYP**: children and young people*

***Supplementary Figure C** – Dataflow diagram for England*

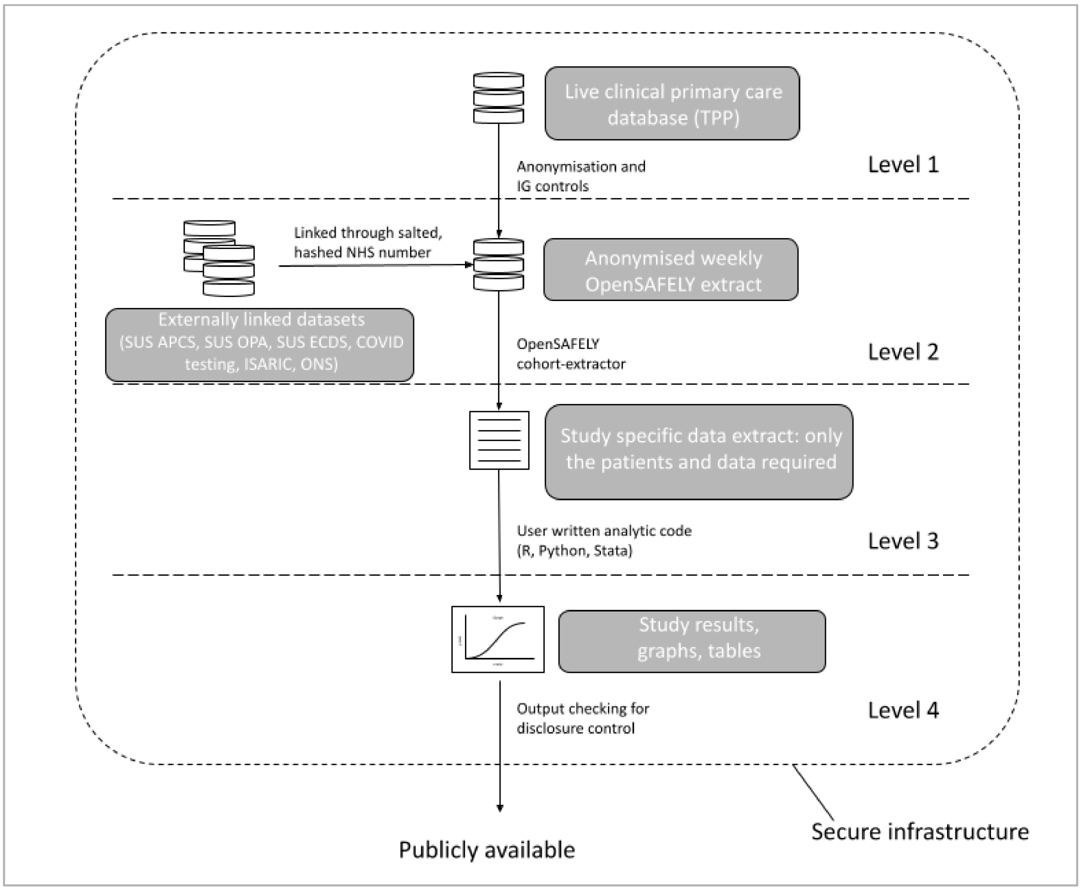

***Abbreviations: TPP:** The Phoenix Partnership (GP group), **SGSS**: Second Generation Surveillance System, **ONS**: Office for National Statistics, **SUS**: Secondary Use Services, **APCS**: Admitted patient care statistics, **OPA**: Outpatient attendances, **ECDS**: Emergency care datasets, **ONS**: Office for National Statistics.*

Swann OV, *et al. BMJ Open* 2022; 12:e063271. doi: 10.1136/bmjopen-2022-063271

Supplementary Figure D– *Directed acyclic graph factors associated with SARS-CoV-2 RT-PCR testing and healthcare use*

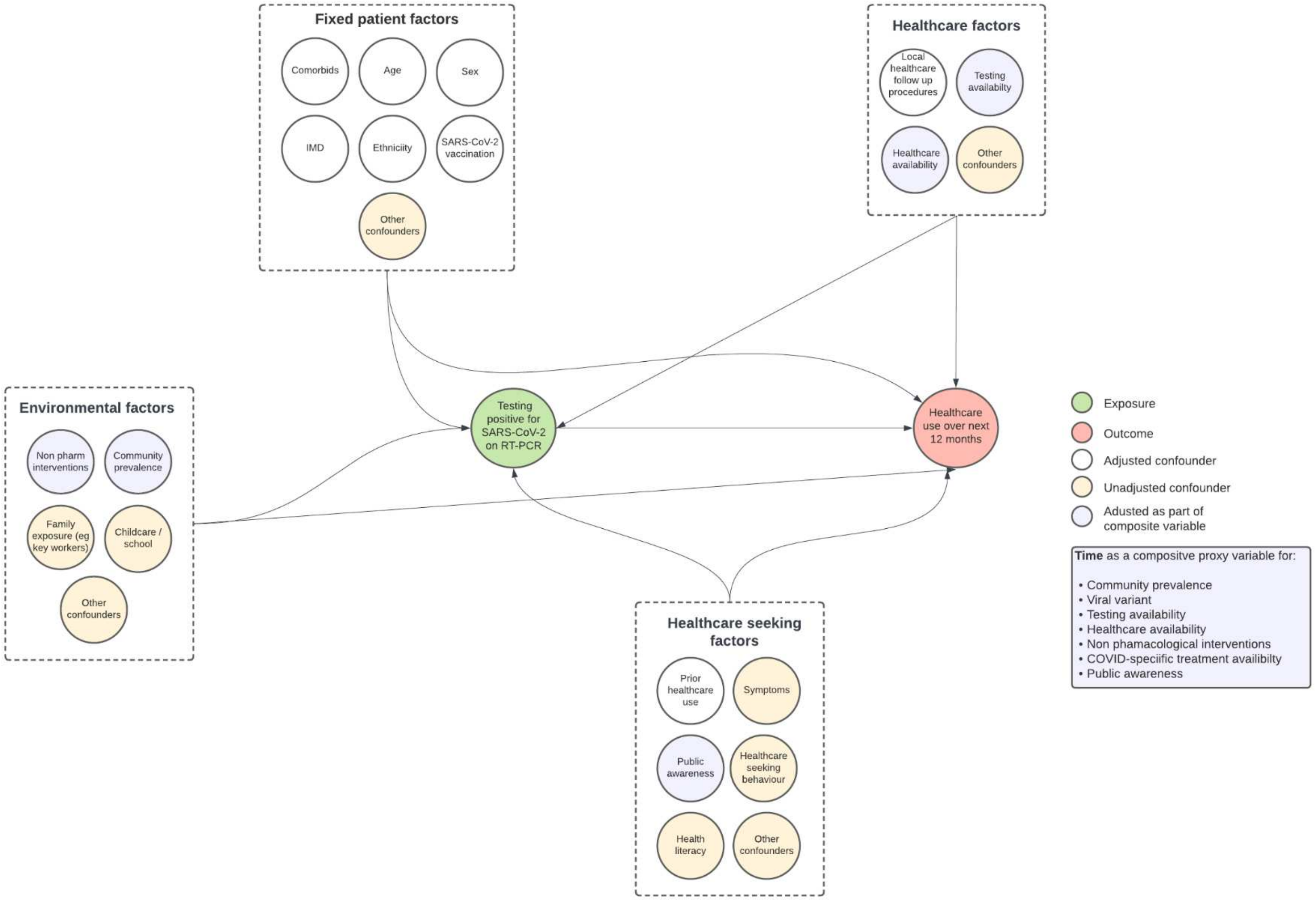

***Supplementary Figure E**. Graphical illustration of potential study scenarios with population controls (Objective 2 sensitivity analysis)*

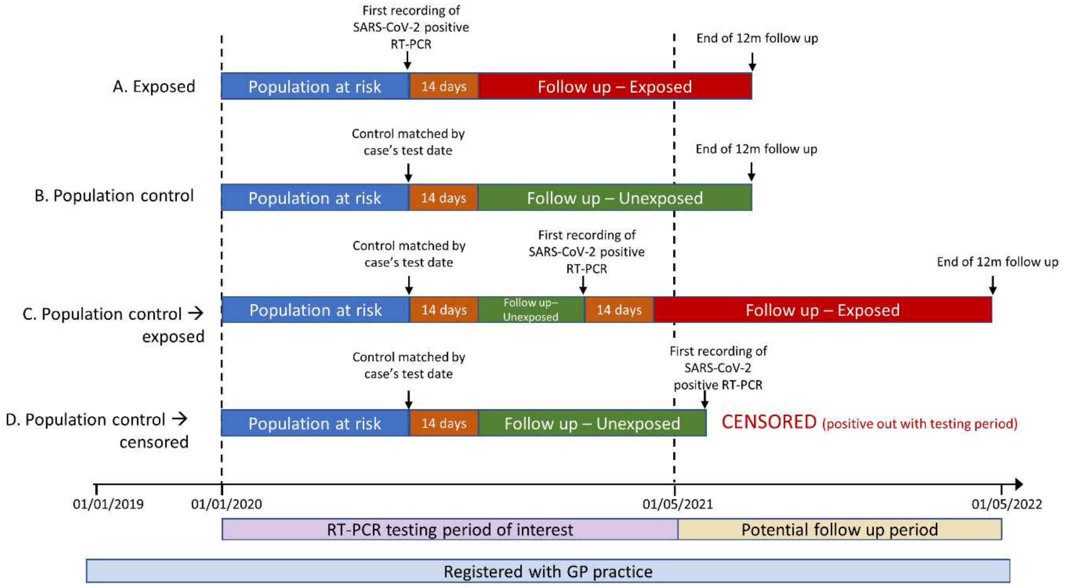

Example A: Positive SARS-CoV-2 RT-PCR case. **Individual A** is followed-up from 14 days after SARS-CoV-2 infection for 12 months. **Examples B-D: Population controls. Individual B** is matched to an individual with SARS-CoV-2 infection and followed-up from 14 days after matching for 12 months. **Individual C** is matched to an individual with SARS-CoV-2 infection and followed-up from 14 days after matching until they are first recorded with SARS-CoV-2 infection themselves during the RT-PCR testing period of interest. At this point they are censored from further follow-up as a test negative comparator and followed-up as an exposed case from 14 days after infection for 12 months with appropriate matches for the date of positive RT-PCR. **Individual D** is matched to an individual with SARS-CoV-2 infection and followed-up from 14 days after matching until they are first recorded with SARS-CoV-2 infection themselves. As this occurs after the RT-PCR testing period of interest, they are censored from further follow-up as an unexposed comparator.

Supplementary Figure F – Logo for SLICK study

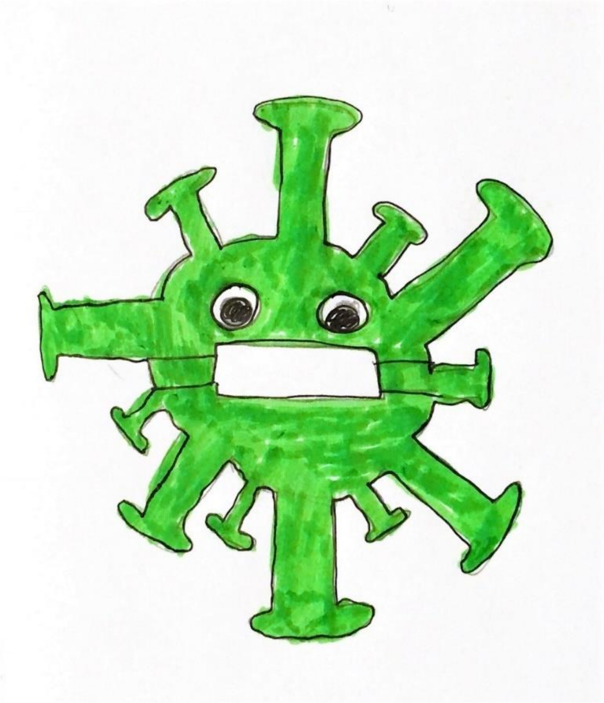

Designed by Georgia Langley, aged 11 (consent obtained from parent to publish child's name)

OpenSAFELY Information governance and ethical approval – further information

NHS England is the data controller for OpenSAFELY-TPP; TPP is the data processor; all study authors using OpenSAFELY have the approval of NHS England. This implementation of OpenSAFELY is hosted within the TPP environment which is accredited to the ISO 27001 information security standard and is NHS IG Toolkit compliant. [1]

Patient data has been pseudonymised for analysis and linkage using industry standard cryptographic hashing techniques; all pseudonymised datasets transmitted for linkage onto OpenSAFELY are encrypted; access to the platform is via a virtual private network (VPN) connection, restricted to a small group of researchers; the researchers hold contracts with NHS England and only access the platform to initiate database queries and statistical models; all database activity is logged; only aggregate statistical outputs leave the platform environment following best practice for anonymisation of results such as statistical disclosure control for low cell counts.[2]

The OpenSAFELY research platform adheres to the obligations of the UK General Data Protection Regulation (GDPR) and the Data Protection Act 2018. In March 2020, the Secretary of State for Health and Social Care used powers under the UK Health Service (Control of Patient Information) Regulations 2002 (COPI) to require organisations to process confidential patient information for the purposes of protecting public health, providing healthcare services to the public and monitoring and managing the COVID-19 outbreak and incidents of exposure; this sets aside the requirement for patient consent.[3] This was extended in July 2022 for the NHS England OpenSAFELY COVID-19 research platform.[4] In

some cases of data sharing, the common law duty of confidence is met using, for example, patient consent or support from the Health Research Authority Confidentiality Advisory Group.[5]

Taken together, these provide the legal bases to link patient datasets on the OpenSAFELY platform. GP practices, from which the primary care data are obtained, are required to share relevant health information to support the public health response to the pandemic, and have been informed of the OpenSAFELY analytics platform.

Supplementary references:

1. Data Security and Protection Toolkit - NHS Digital. NHS Digital. https://digital.nhs.uk/data-and-information/looking-after-information/data-security-and-information-governance/data-security-and-protection-toolkit (accessed 30 Apr 2020).

2. ISB1523: Anonymisation Standard for Publishing Health and Social Care Data - NHS Digital. NHS Digital. https://digital.nhs.uk/data-and-information/information-standards/information-standards-and-data-collections-including-extractions/publications-and-notifications/standards-and-collections/isb1523-anonymisation-standard-for-publishing-health-and-social-care-data (accessed 30 Apr 2020).

3. Secretary of State for Health and Social Care - UK Government. Coronavirus (COVID-19): notification to organisations to share information. 2020. https://web.archive.org/web/20200421171727/https://www.gov.uk/government/publications/coronavirus-covid-19-notification-of-data-controllers-to-share-information

4. Secretary of State for Health and Social Care - UK Government. Coronavirus (COVID-19): notification to organisations to share information. 2022. https://www.gov.uk/government/publications/coronavirus-covid-19-notification-to-organisations-to-share-information/coronavirus-covid-19-notice-under-regulation-34-of-the-health-service-control-of-patient-information-regulations-2002

5. Confidentiality Advisory Group. Health Research Authority. https://www.hra.nhs.uk/about-us/committees-and-services/confidentiality-advisory-group/
