# Supplementary material for: Studying the Long-term Impact of COVID-19 in Kids (SLICK). Healthcare use and costs in children and young people following community-acquired SARS-CoV-2 infection: protocol for an observational study using linked primary and secondary routinely collected healthcare data from England, Scotland and Wales
Source: BMJ Open. 2022 Nov 10;12(11):e063271. doi: 10.1136/bmjopen-2022-063271 (PMC9659708; doi:10.1136/bmjopen-2022-063271)
Supplement: Author's manuscript [file bmjopen-2022-063271.draft_revisions.pdf]

BMJ Open is committed to open peer review. As part of this commitment we make the peer review history of every article we publish publicly available.

When an article is published we post the peer reviewers' comments and the authors' responses online. We also post the versions of the paper that were used during peer review. These are the versions that the peer review comments apply to.

The versions of the paper that follow are the versions that were submitted during the peer review process. They are not the versions of record or the final published versions. They should not be cited or distributed as the published version of this manuscript.

BMJ Open is an open access journal and the full, final, typeset and author-corrected version of record of the manuscript is available on our site with no access controls, subscription charges or pay-per-view fees (http://bmjopen.bmj.com).

If you have any questions on BMJ Open's open peer review process please email info.bmjopen@bmj.com

BMJ Open

Studying the Long-term Impact of Covid in Kids (SLICK). Healthcare use and costs in children and young people following community-acquired SARS-CoV-2 infection: protocol for an observational study using linked primary and secondary routinely collected healthcare data from England, Scotland and Wales.

Journal:	*BMJ Open*
Manuscript ID	bmjopen-2022-063271
Article Type:	Protocol
Date Submitted by the Author:	05-Apr-2022

Complete List of Authors:	Swann, Olivia; The University of Edinburgh Usher Institute of Population Health Sciences and Informatics, Centre for Medical Informatics; The University of Edinburgh Department of Child Life and Health,<br>Lone, Nazir; The University of Edinburgh Usher Institute of Population Health Sciences and Informatics, Centre for Population Health Sciences<br>Harrison, Ewen; The University of Edinburgh Usher Institute of Population Health Sciences and Informatics, Centre for Medical Informatics<br>Tomlinson, Laurie; London School of Hygiene and Tropical Medicine Department of Non-communicable Disease Epidemiology<br>Walker, Alex; University of Oxford Nuffield Department of Primary Care Health Sciences, The DataLab<br>Seaborne, Michael; Swansea University, Centre for Population Health<br>Pollock, Louisa; University of Glasgow Department of Child Health<br>Farrell, James; The University of Edinburgh Usher Institute of Population Health Sciences and Informatics, Centre for Medical Informatics<br>Hall, Peter; The University of Edinburgh MRC Institute of Genetics and Molecular Medicine, Institute of Cancer and Genetics<br>Seth, Sohan; The University of Edinburgh School of Informatics<br>Williams, Thomas; The University of Edinburgh Department of Child Life and Health,<br>Preston, Jenny; University of Liverpool Faculty of Humanities and Social Sciences<br>Ainsworth, Jennifer; University of Liverpool Faculty of Humanities and Social Sciences<br>Semple, Freya; University of Glasgow School of Medicine Dentistry and Nursing<br>Baillie, J; The University of Edinburgh The Roslin Institute, Division of Genetics and Genomics<br>Katikireddi, Srinivasa; University of Glasgow, MRC/CSO Social & Public Health Sciences Unit<br>Akbari, Ashley; Swansea University Medical School; Swansea University Medical School, Health Data Research UK<br>Lyons, Ronan; Swansea University Medical School<br>Simpson, Colin; The University of Edinburgh, Usher Institute of

Population Health Sciences and Informatics
Semple, Malcolm G; NIHR Health Protection Research Unit in Emerging and Zoonotic Infections at University of Liverpool; Alder Hey Children's Hospital, Respiratory Paediatrics
Goldacre, Ben; University of Oxford Nuffield Department of Primary Care Health Sciences, The DataLab
Brophy, Sinead; Swansea University Medical School, Health Data Research UK
Sheikh, Aziz; The University of Edinburgh Usher Institute of Population Health Sciences and Informatics
Docherty, Annemarie; The University of Edinburgh Usher Institute of Population Health Sciences and Informatics, Centre for Medical Informatics; University of Edinburgh, Anaesthesia and Critical Care

Keywords: COVID-19, Epidemiology < INFECTIOUS DISEASES, Paediatric infectious disease & immunisation < PAEDIATRICS, HEALTH ECONOMICS

SCHOLARONE™
Manuscripts

Studying the Long-term Impact of COVID-19 in Kids (SLICK). Healthcare use and costs in children and young people following community-acquired SARS-CoV-2 infection: protocol for an observational study using linked primary and secondary routinely collected healthcare data from England, Scotland and Wales.

For peer review only

Author list and affiliations

Olivia V. Swann [1, 2 *], Nazir Lone [3], Ewen M. Harrison [1], Laurie A. Tomlinson [4], Alex J. Walker [5], Michael Seaborne [6], Louisa Pollock [7], James Farrell [1], Peter S Hall [8], Sohan Seth [9], Thomas C. Williams [2], Jennifer Preston [10], J. Samantha Ainsworth [10], Freya F. Semple [11], J. Kenneth Baillie [12], Srinivasa V Katikireddi [13], Ashley Akbari [14], Ronan Lyons [14], Colin R Simpson [3, 15], Malcolm G. Semple [16], Ben Goldacre [5], Sinead Brophy [17], Aziz Sheikh [3] and Annemarie B. Docherty [1]

* *Corresponding author*: Dr Olivia Swann, PhD, Clinical Lecturer in Paediatric Infectious Diseases, Centre for Medical Informatics, Usher Institute, University of Edinburgh, Edinburgh, UK.

Email: Olivia.Swann@ed.ac.uk

Affiliations

1. Centre for Medical Informatics, Usher Institute, University of Edinburgh, Edinburgh, UK
2. Department of Child Life and Health, University of Edinburgh, Edinburgh, UK
3. Usher Institute, University of Edinburgh, Edinburgh, UK
4. Department of Non-Communicable Disease Epidemiology, London School of Hygiene and Tropical Medicine, London, United Kingdom.

5. The DataLab, Nuffield Department of Primary Care Health Sciences, University of Oxford, Oxford, UK
6. Centre for Population Health, Swansea University, Swansea, UK
7. Child Health, School of Medicine, Dentistry and Nursing, University of Glasgow, Glasgow, UK
8. Institute of Cancer and Genetics, University of Edinburgh, Edinburgh, UK
9. School of Informatics, University of Edinburgh, Edinburgh, UK
10. Faculty of Humanities & Social Sciences, University of Liverpool
11. School of Medicine, Dentistry and Nursing, University of Glasgow, Glasgow, UK
12. Roslin Institute, University of Edinburgh, Edinburgh, UK
13. MRC/CSO Social & Public Health Sciences Unit, University of Glasgow, Glasgow, UK
14. Swansea University Medical School, Swansea University, Swansea, UK
15. School of Health, Faculty of Health, Victoria University of Wellington, Wellington, New Zealand
16. NIHR Health Protection Research Unit in Emerging and Zoonotic Infections and Institute of Infection, Veterinary and Ecological Sciences, Faculty of Health and Life Sciences, University of Liverpool, Liverpool, UK
17. Health Data Research UK, Swansea University Medical School, Swansea University, Swansea, UK.

For peer review only

Abstract

Introduction

Severe acute respiratory syndrome coronavirus 2 (SARS-CoV-2) infection rarely causes hospitalisation in children and young people (CYP), but mild or asymptomatic infections are common. Persistent symptoms following infection have been reported in CYP but subsequent healthcare use is unclear. We aim to describe healthcare use in CYP following community-acquired SARS-CoV-2 infection and identify those at risk of ongoing healthcare needs.

Methods and analysis

We will use anonymised individual-level, population-scale national data linking demographics, comorbidities, primary and secondary care use, mortality and SARS-CoV-2 test data between 01/01/2019-01/05/2022. Analyses will use Trusted Research Environments: OpenSAFELY in England, Secure Anonymised Information Linkage (SAIL Databank) in Wales and Early Pandemic Evaluation and Enhanced Surveillance of COVID-19 (EAVE-II) in Scotland. CYP aged ≥4 and <18 years who underwent SARS-CoV-2 reverse transcription polymerase chain reaction (RT-PCR) testing between 01/01/20 and 01/05/21 and those untested CYP will be examined.

The primary outcome measure is cumulative healthcare cost over 12 months following SARS-CoV-2 testing, stratified into primary or secondary care, and physical or mental healthcare. We will estimate the burden of healthcare use attributable to SARS-CoV-2 infections in the 12 months after testing using a matched cohort study of RT-PCR positive, negative or untested CYP matched on testing date, with adjustment for confounders. We will identify factors associated with higher healthcare needs in the 12 months following SARS-CoV-2 infection using an unmatched cohort of RT-PCR positive CYP. Multivariable logistic regression and machine learning approaches will identify risk factors for high healthcare use and characterise patterns of healthcare use post infection.

Ethics and dissemination

This study was approved by the South-Central Oxford C Health Research Authority Ethics Committee (13/SC/0149). Findings will be pre-printed and published in peer-reviewed

For peer review only

journals. Analysis code and code-lists will be available through public GitHub repositories and OpenCodelists with meta-data via HDR-UK Innovation Gateway.

For peer review only

Strengths and limitations of this study

Strengths:

1. Objective, direct examination of clinician-recorded healthcare use by children and young people (CYP) post SARS-CoV-2 infection with population-wide coverage of all CYP <18 years in Scotland and Wales and approximately 4.8 million CYP in England.
2. Reduction in selection and response biases present in much of the existing literature examining persistent symptoms post SARS-CoV-2 infection in CYP.

Limitations:

1. Lack of access to SARS-CoV-2 lateral flow testing results may result in misattribution of SARS-CoV-2 status in patients when reverse transcription polymerase chain reaction (RT-PCR) testing was not performed.
2. Access to health services is presumed to be available for anyone who needed it, but this may have been reduced by local healthcare policies and patient health-seeking behaviour at different points during the pandemic.
3. Owing to the time needed for 12 months of follow up, this study will focus on healthcare use after infection with wildtype and Alpha variants of SARS-CoV-2, which may differ from Delta and Omicron.

For peer review only

Introduction

Severe acute respiratory syndrome coronavirus 2 (SARS-CoV-2) causes the disease COVID-19, with adults being more severely affected than children throughout the pandemic [1]. While hospitalisation with SARS-CoV-2 is rare in children and young people (CYP) [2], infection is common, with up to 70% (95% CI 68-71) of 5-14 year olds estimated to have been infected with SARS-CoV-2 in the UK by December 2021 [3]. Whilst research on COVID-19 in CYP has focused on index hospitalisations and deaths, this acute view means we have not established what the additional healthcare needs are for the majority of CYP after mild or asymptomatic SARS-CoV-2 infection. There is also little information on the changes to healthcare use in children with co-morbidities who may be at risk of exacerbations (for example asthma). The large numbers of CYP infected with SARS-CoV-2 in the UK means that even a small increase in healthcare use in this population could substantially impact on healthcare services. Being asymptomatic with initial infection does not guarantee against developing subsequent illness from SARS-CoV-2, for example CYP who are asymptomatic with their initial SARS-CoV-2 infection can develop Multisystem Inflammatory Syndrome in Children (MIS-C) two to eight weeks later [4]. Whilst this complication is extremely rare (approximately 3 cases per 10,000 infections [5]), it underlines the need to include CYP who are initially asymptomatic from SARS-CoV-2 infection when examining subsequent healthcare use.

A wide variety of persistent symptoms have been reported in CYP following SARS-CoV-2 infection with studies varying in design and quality (reviewed in [6]). Most reports have used a questionnaire or clinic-based approach to symptom reporting, often after hospitalisation with COVID-19 or in patients self-identifying as having Long-COVID, introducing significant potential sources of bias. Whilst adult studies have reported increased risk of outpatient healthcare use in the six months following SARS-CoV-2 infection [7], there is a lack of studies examining healthcare use in CYP following SARS-CoV-2 infection at a population level. Using routinely collected anonymised electronic health record (EHR) data at an individual-level, population-scale matched by SARS-CoV-2 RT-PCR status to examine healthcare use after SARS-CoV-2 infection in CYP will significantly reduce many of the biases seen in studies to date.

For peer review only

In addition to traditional epidemiological approaches, machine learning methods are also proving increasingly important in the analysis of large routinely collected healthcare datasets in SARS-CoV-2 [8]. Using machine learning to identify clusters of patients with similar healthcare trajectories provides a complementary approach to traditional epidemiology to identify patients at risk of high healthcare use post infection. A combination of approaches would establish the long-term healthcare use attributable to SARS-CoV-2 in CYP, which is essential both for tailoring individual care for patients at risk of high healthcare use post infection and informing health service and vaccination planning.

Aims

We aim to establish the patterns and burden of healthcare use in CYP attributable to community-acquired SARS-CoV-2 infection and identify those CYP at risk of high or ongoing healthcare needs in England, Scotland and Wales.

Objectives

We will:

1. Describe the background healthcare use in CYP before and during the pandemic.
2. Compare healthcare use in CYP in the 12 months after testing positive, negative or not being tested for SARS-CoV-2 by RT-PCR to estimate burden of healthcare use attributable to SARS-CoV-2.
3. Identify factors associated with higher healthcare use (including having co-morbidities) in the 12 months following SARS-CoV-2 infection.

For peer review only

Methods

Study period

The period covered by the study will span 01/01/2019 to 01/05/22 and focus on SARS-CoV-2 infections until 01/05/21. This study period was chosen to provide 12 months of follow up data for CYP infected to the end of the second wave of SARS-CoV-2 in the UK (end of April 2021 [9]) as well as those testing negative or not tested. Inclusion of the time frame 01/01/19 to 01/01/21 will provide at least a year of data on pre-pandemic data on healthcare use for each CYP.

Study design

The study will comprise three main approaches; a descriptive graphical analysis addressing Objective 1 (background healthcare use before and during the pandemic), a matched cohort study addressing Objective 2 (estimating healthcare use post SARS-CoV-2 infection) and an unmatched cohort study addressing Objective 3 (identifying factors associated with higher healthcare needs post SARS-CoV-2 infection).

Study Population

The study population will vary with objective:

Inclusion criteria (all objectives):

- Registered with a General Practitioner (GP) in Scotland (includes all general practices), Wales or England (The Phoenix Partnership (TPP) a group of GP practices with a unified electronic patient-record system covering approximately 34% of practices in England [10])

Exclusion criteria (all objectives):

- Positive index SARS-CoV-2 RT-PCR test performed after 7 days in hospital (to exclude nosocomial infections [11])
- CYP with discrepant SARS-CoV-2 RT-PCR results on the same date

Objective 1- Objective-specific inclusion criteria

- Age ≥4 years and <18 years on 01/01/19 (pre-pandemic period)
- Age ≥4 years and <18 years on 01/01/20 (pandemic period)
- Age ≥4 years and <18 years on 01/01/21 (pandemic period)

For peer review only

Objective 2 - Objective-specific inclusion criteria

- Underwent SARS-CoV-2 PCR testing (or untested but matched to CYP who had been tested) between 01/01/20 and 01/05/21
- Age ≥4 and <18 years on date of testing / matching
- At least 12 months of healthcare data available both before and after SARS-CoV-2 PCR test / date of matching if not tested
- No previous positive SARS-CoV-2 PCR test recorded

Objective 3 - Objective-specific inclusion criteria

- Positive SARS-CoV-2 RT-PCR test between 01/01/20 and 01/05/21
- Age ≥4 and <18 years on date of testing
- At least 12 months of healthcare data available both before and after SARS-CoV-2 PCR test
- No previous positive SARS-CoV-2 PCR test recorded

For peer review only

Data sources and validation

Data will be held securely and analyses conducted within nation-specific Trusted Research Environments (TREs): OpenSAFELY in England [12], Secure Anonymised Information Linkage (SAIL Databank [13]) in Wales and the Early Pandemic Evaluation and Enhanced Surveillance of COVID-19 (EAVE-II) platform [14] within Public Health Scotland in Scotland.

Deterministic and probabilistic linking of datasets will be carried out via Community Health Index (CHI) number in Scotland and by National Health Service (NHS) number in England and Wales. NHS and CHI numbers are unique identifiers used in all health-care contacts across the NHS [15]. Datasets contributing to each country's final database are described in *Supplementary Table 1* with data flow diagrams in *Supplementary Figures A-C*. In addition to the study period outlined, data from birth will also be examined to identify comorbidities, including common chronic childhood conditions [16]. In the event of missing data, these will be supplemented by information for that CYP in linked datasets. All variables will be checked for patterns of missingness and implausible values and a log maintained for reasons where records are excluded from analysis. In cases where an analysis variable has high levels of missingness, alternative variables which are closely related may be considered as a proxy for these missing data. Depending on the cause ascertained for missing variables, we will

consider imputation.

As PIMS-TS is a new disease, ICD-10 coding was not introduced until November 2020. Admission will be considered due to PIMS-TS if occurring between 01/02/20 – 01/11/20 and coded as Kawasaki disease, toxic shock syndrome or systemic inflammatory response (proxies for PIMS-TS) or if admitted after 01/11/20 and coded as PIMS-TS [17]. National PIMS-TS databases (available in Scotland and Wales) will be used for sensitivity analyses. The major data sources for each variable are detailed in *Table 1*.

For peer review only

Table 1. Groupings of variables by source (adapted from [18])

	Variable	Data source		
		**England (OpenSAFELY)**	**Scotland (EAVE-II)**	**Wales (SAIL)**
**Demographics**	Sex<br>Age<br>Ethnicity	TPP<br>TPP<br>TPP	EAVE-II<br>EAVE-II<br>EAVE-II	WDSD/WLGP<br>WDSD/WLGP<br>CENW/NCCH
**Socio-economic**	IMD	TPP	EAVE-II	WDSD
**Place of residence**	Health board / STP, urban rural index	TPP	EAVE-II	WDSD
**Accommodation type**	Private or social housing	*NA*	EAVE-II	CENW
**Comorbidities**	Chronic childhood conditions<br>SARS-CoV-2 shielding list	TPP, SUS APCS, SUS OPA, ISARIC<br>TPP	EAVE-II, SMR00/01/04/06, ISARIC<br>EAVE-II	CYFI, BREC, WCSU, WLGP, PEDW<br>CVSP
**SARS-CoV-2 vaccination**	Vaccine (type, date)	TPP	TVMT	CVVD
**Laboratory tests**	RT-PCR SARS-CoV-2 test (date and result)<br>Viral variant	SGSS<br>SGSS	COVID-testing<br>COG UK	PATD<br>CVSD/PATD
**Secondary care**	ED contact<br>Outpatient clinic contact<br>Hospital admission<br>Admission ICD-10 code<br>Level of care<br>Length of stay<br>PIMS-TS	SUS ECDS<br>SUS OPS<br>SUS APCS<br>SUS APCS<br>SUS / ISARIC<br>SUS / ISARIC<br>SUS / ISARIC	A+E Datamart<br>SMR00<br>SMR01/04<br>SMR<br>SMR / ISARIC<br>SMR / ISARIC<br>SMR / ISARIC	EDDD/EDDS<br>OPDW<br>PEDW<br>PEDW<br>PEDW/CCDS<br>PEDW/CCDS<br>PEDW/CCDS
**Primary care**	In-hours contact<br>Community prescriptions	TPP<br>TPP	EAVE-II<br>EAVE-II / PIS	WLGP<br>WDDS
**Unscheduled care**	NHS 111 contact<br>Ambulance contact<br>GP out of hours contact	*NA*<br>*NA*<br>*NA*	NHS 24<br>SAS<br>GP OOH	NHSO<br>WASD/NHSO<br>NHSO
**Mortality**	Death (all cause, COVID-19 main cause or <28 days of positive SARS-CoV-2 RT-PCR)	ONS deaths	NRS deaths	ONS deaths / ADDE

For peer review only

**Symptoms**	Presenting symptoms in CYP admitted with SARS-CoV-2	ISARIC (subset only)	ISARIC (subset only)	ISARIC (subset only)

Abbreviations: **EAVE-II=**Early Pandemic Evaluation and Enhanced Surveillance of COVID-19; **SAIL**: Secure Anonymised Information Linkage; **IMD=**Index of Multiple Deprivation; **STP=**Sustainability and Transformation Partnership (STP, geographical areas configured for regional reorganisation in England), **ED=** Emergency Department; **ICD-10=**International Classification of Diseases 10th Revision; **NHS=**National Health Service; **GP=**general practice; **PIMS-TS=** Paediatric multisystem inflammatory syndrome temporally associated with COVID-19; **TPP=**The Phoenix Partnership (GP group); **SUS**=Secondary Use Services; **APCS**=Admitted patient care statistics; **OPA**=Outpatient attendances; **ECDS**=Emergency care datasets; **SGSS**=Second Generation Surveillance System; **ISARIC** =International Severe Acute Respiratory and emerging Infection Consortium / COVID-19 Clinical Information Network; **ONS**: Office for National Statistics; **SMR**=Scottish Morbidity Record; **TMVT**=Turas Vaccination Management Tool; **COG-UK**=Centre of Genomics United Kingdom; **PIS**= Prescribing Information System; **SAS**=Scottish Ambulance Service; **OOH**=Out Of Hours; **NRS**=National Records of Scotland; **WLGP**=Welsh Longitudinal General Practice; **PEDW**=Patient Episode Database for Wales; **ADDE**=Annual District Death Extract; **CCDS=**Critical Care Data Source; **CDDS=**COVID-19 Consolidated Deaths; **CENW=**Office of National Statistics Census; **CTTP=**COVID-19 Test Trace & Protect; **CVLF=**COVID-19 Lateral Flow; **CVSP=**COVID-19 Shielded People; **CVVD=**COVID-19 Vaccine Data; **EDDD=**Emergency Department Dataset Daily; **EDDS=**Emergency Department Dataset; **ICCD=**Intensive Care National Audit & Research Centre (ICNARC)-COVID only admissions; **ICNC=**Intensive Care National Audit & Research Centre (ICNARC); **MIDS=**Maternity Indicators Dataset; **NCCH=**National Community Child Health; **NHSO=**NHS 111 Call data; **OPDW=**Outpatient Dataset for Wales; **OPRD=**Outpatient Referral Dataset; **PATD=**Pathology Data (COVID-19 daily); **RTTD=**Referral to Treatment Times Dataset; **WASD=**Welsh Ambulance Service Dataset; **WCSU=**Welsh Cancer Incidence Surveillance Unit; **WDDS=**Welsh Dispensing Dataset; **WDSD=**Welsh Demographic Service Dataset. **NA**=Not available.

For peer review only

Exposure

The exposure of interest is diagnosis of SARS-CoV-2 infection, defined as a positive RT-PCR test result. The date of exposure is defined as the date of the positive RT-PCR test result.

Outcomes

The primary outcome measure will be cumulative NHS healthcare costs over the 12 months following SARS-CoV-2 testing. This will provide an overarching measure that is reflective of healthcare resource use, which is expressed on a monetary scale that is common between the three nations and common to all types of activity. Activity will only contribute to the primary outcome measure if it is quantifiable from data in all three nations. Healthcare costs will be broken down into budget-holder perspectives; secondary care (critical care/inpatient/outpatient/A&E) and primary care (face-to face or telephone in-hours primary care activity). A sensitivity analysis of unscheduled care (e.g. NHS 24, ambulance, GP OOH) will be undertaken for the nations where this data is available (Scotland and Wales). To ensure comparability, unit costs will be assigned from a common country (England) using Personal Social Services Research Unit costs with a common base year [19].

Secondary outcomes will constitute units of healthcare activity, quantifiable as counts over time or rates, that can be quantified to a common definition between the three nations, e.g. inpatient episodes by specialty or primary care appointments. Both primary and secondary outcomes will be stratified into predominantly physical or mental healthcare based on the primary reason for admission / attendance. The reason for healthcare use will also be further explored (e.g. by body system / healthcare speciality).

For peer review only

Statistical analyses

Analyses will be replicated across the three nations in each respective TRE.

Objective 1

Describe the background healthcare use in CYP before and during the pandemic.

Significant, dynamic changes in both healthcare access and healthcare-seeking behaviour have occurred across the course of the pandemic to date. As such, exploration of background healthcare use in CYP before and during the pandemic will help contextualise subsequent analyses. A descriptive, graphical analysis will be undertaken. Healthcare use (represented as cost) will be plotted for the period of 01/01/19 to 01/05/22 for all CYP. These data will be stratified by variables including age, sex, nation of residence, type of healthcare (primary or secondary care) and RT-PCR status (RT-PCR positive, RT-PCR negative and never tested). Reasons for healthcare visits will also be explored.

Objective 2

Compare healthcare use in CYP in the 12 months after testing positive, negative or not being tested for SARS-CoV-2 by RT-PCR to estimate the burden of healthcare use attributable to SARS-CoV-2.

This analysis will focus on estimating the burden of CYP healthcare use which is attributable to SARS-CoV-2 infection in the 12 months after infection, whereas individual factors associated with healthcare use after infection will be explored in Objective 3.

A prospective matched cohort study will be undertaken. Matching will be undertaken for date of RT-PCR test with iterative widening bands as necessary. This will account for availability of testing, access to healthcare, variation in incidence rates, emergence of viral variants, changes in SARS-CoV-2 treatment and systematically different characteristics in the tested population (compared to the untested population) as the pandemic progressed. Ten RT-PCR test negative non-hospitalised control CYP will be matched without replacement for every RT-PCR positive case. Confounding will then be minimised by propensity score development and / or adjustment including the following variables: age, sex, SARS-CoV-2 vaccination status at the time of index RT-PCR test (considered vaccinated if ≥3 weeks since first dose), geographical region (health board / Sustainability and Transformation

For peer review only

Partnership (STP)) to account for regional differences in RT-PCR testing and availability of healthcare, previous healthcare contact (primary or secondary), chronic conditions, number of previous SARS-CoV-2 tests, socioeconomic status (quintiles of relevant national deprivation measure: Scottish Index of Multiple Deprivation (SIMD), Welsh Index of Multiple Deprivation (WIMD) and Lower layer Super Output Area (LSOA)) and urban-rural index.

Factors are associated with being brought for RT-PCR testing (e.g. public awareness and testing availability) may be different from those of exposure to SARS-CoV-2. A directed acyclic graph of factors associated with SARS-CoV-2 RT-PCR testing and healthcare use to consider in model building is shown in *Supplementary Figure D.*

In contrast to adults, the median hospital length of stay due to SARS-CoV-2 in CYP is short, previously reported in the UK as 2 days (IQR 1-4) [20]. As such, follow up will start 14 days after testing positive for SARS-CoV-2 on RT-PCR which will enable us to look back and further stratify the exposure by SARS-CoV-2 severity (i.e. community care, hospitalisation or critical care).

CYP in the control group may subsequently test positive for SARS-CoV-2 by RT-PCR. If this occurs during the RT-PCR testing period of interest (01/01/20 - 01/05/21) they will become a case and follow-up commenced for 12 months (with appropriate matches for the date of the positive RT-PCR). If the control tests positive after 01/05/21 (i.e. after the RT-PCR testing period of interest), they will be censored and will not become a case. A graphical illustration of the potential CYP paths for this analysis is shown in *Figure 1.*

As CYP who are brought for RT-PCR testing are systematically different to those who are not brought [21], a sensitivity analysis will be undertaken to compare the RT-PCR positive cohort against the population of CYP who have never tested positive (i.e. both RT-PCR negative and untested CYP), hereafter "population controls." RT-PCR positive CYP will be matched to ten population controls who were not hospitalised on the date of their matched case's RT-PCR [7]. Confounding will then be minimised as described above. A graphical illustration of the potential CYP paths for this analysis is shown in *Supplementary Figure E.*

The proportion of CYP with SARS-CoV-2 infection but without a positive RT-PCR (e.g. tested by lateral flow or untested asymptomatic cases) has increased across the pandemic [3].

For peer review only

As such, we will conduct quantitative bias analyses for unmeasured confounding using different estimates of undetected SARS-CoV-2 infection across the study period.

Objective 3

Identify factors associated with higher healthcare use (including having co-morbidities) in the 12 months following SARS-CoV-2 infection.

Both regression and machine learning approaches will be undertaken to examine healthcare costs in the SARS-CoV-2 RT-PCR positive cohort. A multivariable regression model will be constructed with covariates including demographics (age, sex, socioeconomic status, urban-rural Index and health board / STP, pre-existing health status (chronic comorbidities, previous health care resource use, number of dispensed prescriptions, vaccination status and number of previous PCR tests), markers of severity of illness (community, hospital or intensive care within 14 days of index RT-PCR positive result) and PIMS-TS. In order to examine CYP admitted due to SARS-CoV-2 (rather than those with incidental SARS-CoV-2 infection and another reason for admission), a sensitivity analysis will be performed excluding CYP with index SARS-CoV-2 RT-PCR undertaken 72 hours or less before an elective admissions, day case procedure or undertaken at any time during hospitalisation for trauma or emergency surgery.

We will then explore machine learning approaches to identify patterns of healthcare use over time following SARS-CoV-2 infection and explore which covariates are associated with high healthcare use. We will categorise CYP into groups based on their trajectories (i.e. patterns of healthcare use). Both total healthcare cost and types of healthcare (secondary care and scheduled primary care) will be considered. This will be done using three approaches: a) latent growth mixture model of aggregated healthcare uses over a month [22], b) Bayesian categorical time series clustering of daily service uses of different types [23], and c) centroid based clustering with dynamic time warping distance of smoothed healthcare use cost [24]. By modelling this time series of healthcare use, we will group patients into clusters with similar patterns, e.g., one cluster may correspond to CYP who use general practices on a frequent basis but are not admitted to hospital while another cluster may belong to CYP who do not use general practices but attend outpatient clinics regularly.

For peer review only

After identifying CYP clusters, covariates (including demographics, comorbidities and previous healthcare use) will be examined to identify any factors which may associated with higher healthcare needs post SARS-CoV-2. These analyses will be stratified by hospitalisation (i.e. hospital admission within 14 days of index RT-PCR positive result) or community care and by diagnosis of PIMS-TS. A sensitivity analysis excluding CYP with presumed incidental SARS-CoV-2 will be carried out as detailed above.

Sensitivity analysis

It is likely that the majority of healthcare costs will be experienced within the first three months of SARS-CoV-2 infection [25]. Following on from Objectives 2 and 3, we will extend the end date of the cohort to three months before the date of data extraction, and examine healthcare use in the three months following infection with SARS-CoV-2. This will enable us to examine healthcare use with later Delta (B.1.617.2) and Omicron (B.1.1.529) variants.

Anticipated limitations

Whilst this protocol has been carefully developed to reduce bias, there are anticipated limitations due to constraints of the data. Given the study period, it will also only be possible to examine the annual healthcare costs following infections with wildtype or Alpha (B.1.1.7) SARS-CoV-2 variant infections which may not be the same as after Delta (B.1.617.2) or Omicron (B.1.1.529) variant infections. The datasets included do not contain information on SARS-CoV-2 lateral flow testing results which could result in misattribution of SARS-CoV-2 status in patients if RT-PCR testing was not performed. This is likely to particularly affect the later months of the study period where the highly transmissible Omicron variant was widespread and government advice no longer advocated RT-PCR following a positive lateral flow test in some situations [26]. In addition, the study will presume that healthcare services were available for anyone who needed them, but this may have been affected by local healthcare policies and patient health-seeking behaviour at different points during the pandemic.

For peer review only

Patient and Public Involvement

This proposal was developed together with the Liverpool Generation-R Young Person's Advisory Group (YPAG), a group of engaged CYP aged between 12 and 21 years with lived experience of the SARS-CoV-2 pandemic. A member of the YPAG is also a co-investigator and member of the steering committee, helping ensure the study is delivered appropriately and that decisions about study implementation are guided by meaningful PPIE input. We will undertake two interactive workshops with the YPAG to co-create educational materials for use in schools/science fairs. We will also use these workshops to discuss challenges regarding misinformation about SARS-CoV-2, strategies to correctly share information to young people using social media and the use of routine data in research. The YPAG have named the study – "Studying the Long-term Impact of COVID-19 in Kids (SLICK)" and chosen the logo (*Supplementary Figure F*).

For peer review only

Ethics and Dissemination

This study was approved by the South Central - Oxford C - Health Research Authority Research Ethics Committee, approval reference number 13/SC/0149.

The EAVE-II dataset was approved by the National Research Ethics Service Committee, South East Scotland 02 (REC number: 12/SS/0201) and the Public Benefit and Privacy Panel for Health and Social Care (reference number: 1920-0279).

OpenSAFELY is a secure, transparent, open-source software platform for analysis of electronic health records data with all activity publicly logged. The establishment of the OpenSAFELY platform was approved by the Health Research Authority (REC reference 20/LO/0651). The OpenSAFELY research platform adheres to the data protection principles of the UK Data Protection Act 2018 and the EU General Data Protection Regulation (GDPR) 2016.

The Welsh Con-COV research platform was created to determine demographic, socioeconomic and clinical risk factors for infection and mortality of COVID-19, to measure impact of COVID-19 on healthcare utilisation and long-term health, and to enable the evaluation of natural experiments of policy intervention [27]. The project (SAIL 0911) was

approved by the independent Information Governance Review Panel (IGRP). Investigation of the long-term healthcare burden of COVID-19 in children falls under this remit thus Con-COV is approved for use. Approved researchers are also able to access additional information within Con-COV that has been brought to SAIL under the Digital Economy Act (DEA) to Accredited Researchers via the SAIL Databank [28].

Guidelines for the Strengthening the Reporting of Observational Studies in Epidemiology (STROBE) and REporting of studies Conducted using Observational Routinely-collected Data (RECORD) (via the COVID-19 extension) will be followed to report findings from this study. Findings will be presented at international conferences and published in peer-reviewed journals. Reports will also be prepared for policy makers. All analysis code will be made available through a public GitHub repository. In addition, a methods guide to producing harmonised metrics of paediatric healthcare costs across the three nations will be developed with associated code. Code lists to map and classify long term health conditions in paediatric populations in routine primary and secondary care datasets will be made available through OpenCodelists (www.opencodelists.org). Meta-data will be made available via the HDR-UK Innovation Gateway.

Funding

This research is part of the Data and Connectivity National Core Study, led by Health Data Research UK in partnership with the Office for National Statistics and funded by UK Research and Innovation (grant ref MC_PC_20058). This work was also supported by The Alan Turing Institute via ‘Towards Turing 2.0’ EPSRC Grant Funding. The grant period spans 01/11/21 to 30/09/22.

Acknowledgements

The cohorts included in this work have been funded separately as follows:

ISARIC / CO-CIN is supported by grants from the National Institute for Health Research (award CO-CIN-01) and the Medical Research Council (grant MC_PC_19059) and by the National Institute for Health Research Health Protection Research Unit (NIHR HPRU) in Emerging and Zoonotic Infections at University of Liverpool in partnership with Public Health

For peer review only

England (PHE), in collaboration with Liverpool School of Tropical Medicine and the University of Oxford (NIHR award 200907), Wellcome Trust and Department for International Development (215091/Z/18/Z), and the Bill and Melinda Gates Foundation (OPP1209135).

EAVE II is funded by the Medical Research Council [MR/R008345/1] and supported by the Scottish Government. This work is supported by BREATHE—The Health Data Research Hub for Respiratory Health [MC_PC_19004]. BREATHE is funded through 10 the UK Research and Innovation Industrial Strategy Challenge Fund and delivered through Health Data Research UK.

OpenSAFELY is jointly funded by UKRI [COV0076;MR/V015737/1] NIHR and Asthma UK-BLF and the Longitudinal Health and Wellbeing strand of the National Core Studies programme. The OpenSAFELY data science platform is funded by the Wellcome Trust. BG's work on better use of data in healthcare more broadly is currently funded in part by: the Wellcome Trust, NIHR Oxford Biomedical Research Centre, NIHR Applied Research Collaboration Oxford and Thames Valley, the Mohn-Westlake Foundation; all DataLab staff are supported by BG's grants on this work.

SAIL Databank is funded by Health Care Research Wales and the analysis of this work was also funded by Health Care Research Wales through the Centre for Population Health and through Health Data Research Wales/N.Ireland, which receives its funding from HDR UK Ltd (HDR-9006) funded by the UK Medical Research Council, Engineering and Physical Sciences Research Council, Economic and Social Research Council, Department of Health and Social Care (England), Chief Scientist Office of the Scottish Government Health and Social Care Directorates, Health and Social Care Research and Development Division (Welsh Government), Public Health Agency (Northern Ireland), British Heart Foundation (BHF) and the Wellcome Trust. This work was supported by the Con-COV team funded by the Medical Research Council (grant number: MR/V028367/1).

Funders had no role in the study design, collection, analysis, and interpretation of data; in the writing of the report; and in the decision, submit the article for publication.

For peer review only

Author's contributions

OS, NIL, EMH, PSH, SB, MGS, SB, BG and ABD were responsible for conception of this project. OVS, LAT , AJW, MJS, JF, BG, SB and ABD will be responsible for data curation. OVS, NIL, EMH, LAT, AJW, MJS, JF, SS and ABD will be undertaking the analysis for this protocol. OVS, NIL, EMH, JKB, MGS, BG, SB, AS and ABD were responsible for securing funding for this project or its constituent cohorts. OVS, NIL, EMH, LAT, AJW, MJS, LP, JF, PSH, SS, JP, JSA, FFS, SVK, CRS, MGS, SB and ABD designed the analysis plan. OVS and ABD are providing administrative support to this project. LAT, AJW, MS, JP, JA, FFS, JKB, AA, RL, MGS, BG, SB, AS and ABD are providing resources to this project. EMH, LAT, AJW, MJS, SS and BG are providing software for this project. MGS, AS and ABD are providing supervision. EMH, LAT, AJW, MJS, JF and SS will be responsible for data validation. OVS, EMH, AJW, MJS and SS are responsible for data visualisation. OVS, NIL, EMH, LAT, AJW, MJS, LP, PSH, SS and ABD wrote the original draft of this protocol and all authors were involved in the review and editing of this manuscript.

Competing Interests

OVS reports an institutional payment from HDR-UK/Alan Turing for work on this study. **LAT** reports institutional contracts with UKRI, NIHR, MRC, institutional consulting fees from Bayer, support to attend MHRA meetings and unpaid membership of two non-industry funded trial advisory committees. **MS** reports an institutional payment from HDR-UK/Alan Turing for work on this study. **CRS** reports institutional grants from MBIE, HRC and MRC. **SVK** reports funding from NRS, MRC and the Scottish Government Chief Scientist Office. He was co-chair of the Scottish Government's Expert Reference Group on Ethnicity and COVID-19 and a member of the UK Scientific Advisory Group on Emergencies subgroup on ethnicity. **MGS** reports grants from NIHR, MRC and Health Protection Research Unit in Emerging & Zoonotic Infections, University of Liverpool. He also reports a role as Independent external and non-remunerated member of Pfizer's External Data Monitoring Committee for their mRNA vaccine program. He is Chair of Infectious Disease Scientific Advisory Board for Integrum Scientific LLC, Greensboro, NC, USA and director of MedEx Solutions Ltd. He reports minority stock ownership for Integrum Scientific LLC, Greensboro,

For peer review only

NC, USA and majority stock ownership for MedEx Solutions Ltd. He also reports a gift from Chiesi Farmaceutici SPA to his institution of a clinical trial investigational medicinal product without encumbrance and distribution of same to trial sites. He is also a non-remunerated independent member of HMG UK Scientific Advisory Group for Emergencies (SAGE, COVID-19 Response) and HMG UK New Emerging Respiratory Virus Threats Advisory Group (NERVTAG). **SB** has received an institutional payment from HDR-UK/Alan Turing funding UOE Ref: 11563729 for work on this study. She also reports institutional payments from MRC, Welsh Government and NIHR. She is a member of the Population and Systems Medicine MRC board. **AS** reports an institutional payment from HDR-UK/Alan Turing and research grants for EAVE II and BREATHE Hub. He also reports non-remunerated positions on AstraZeneca's Thrombotic Thrombocytopenic Taskforce and Scottish and UK Government Advisory Committees. RAL is a member of the Welsh Government COVID-19 Technical Advisory Group. **BG** has received research funding from HDRUK, the Laura and John Arnold Foundation, the Wellcome Trust, the NIHR Oxford Biomedical Research Centre, the NHS National Institute for Health Research School of Primary Care Research, the Mohn-Westlake Foundation, the Good Thinking Foundation, the Health Foundation, and the World Health Organisation; he also receives personal income from speaking and writing for lay audiences on the misuse of science.

AJW, NIL, EMH, LP, JF, PSH, SS, AA, TCW, JP, JSA, FFS, JKB and **ABD** report no competing interests.

For peer review only

References

1. Docherty AB, Harrison EM, Green CA, et al. Features of 20 133 UK patients in hospital with covid-19 using the ISARIC WHO Clinical Characterisation Protocol: prospective observational cohort study. *BMJ*; 369. Epub ahead of print 22 May 2020. DOI: 10.1136/bmj.m1985.

2. Swann OV, Holden KA, Turtle L, et al. Clinical characteristics of children and young people admitted to hospital with covid-19 in United Kingdom: prospective multicentre observational cohort study. *BMJ*; 370. Epub ahead of print 27 August 2020. DOI: 10.1136/bmj.m3249.

3. Birrel P, Blake J, van Leeuwen E, et al. Report on Nowcasting and Forecasting - 9th December 2021. *MRC Biostatistics Unit*, https://www.mrc-bsu.cam.ac.uk/now-casting/report-on-nowcasting-and-forecasting-9th-december-2021/ (accessed 9 February 2022).

4. Whittaker E, Bamford A, Kenny J, et al. Clinical Characteristics of 58 Children With a Pediatric Inflammatory Multisystem Syndrome Temporally Associated With SARS-CoV-2. *JAMA*. Epub ahead of print 8 June 2020. DOI: 10.1001/jama.2020.10369.

5. Payne AB, Gilani Z, Godfred-Cato S, et al. Incidence of Multisystem Inflammatory Syndrome in Children Among US Persons Infected With SARS-CoV-2. *JAMA Netw Open* 2021; 4: e2116420.

6. Behnood SA, Shafran R, Bennett S, et al. *Persistent Symptoms Following SARS-CoV-2 Infection Among Children and Young People: A Meta-Analysis of Controlled and Uncontrolled Studies*. SSRN Scholarly Paper ID 3940260, Rochester, NY: Social Science Research Network. Epub ahead of print 12 October 2021. DOI: 10.2139/ssrn.3940260.

7. Al-Aly Z, Xie Y, Bowe B. High-dimensional characterization of post-acute sequelae of COVID-19. *Nature* 2021; 594: 259–264.

8. Zoabi Y, Deri-Rozov S, Shomron N. Machine learning-based prediction of COVID-19 diagnosis based on symptoms. *Npj Digit Med* 2021; 4: 1–5.

9. Office for National Statistics. Coronavirus (COVID-19) Infection Survey technical article: waves and lags of COVID-19 in England, June 2021, https://www.ons.gov.uk/peoplepopulationandcommunity/healthandsocialcare/conditionsa

For peer review only

nddiseases/articles/coronaviruscovid19infectionsurveytechnicalarticle/wavesandlagsofcovid19inenglandjune2021 (2021, accessed 17 December 2021).

10. Kontopantelis E, Stevens RJ, Helms PJ, et al. Spatial distribution of clinical computer systems in primary care in England in 2016 and implications for primary care electronic medical record databases: a cross-sectional population study. *BMJ Open* 2018; 8: e020738.

11. Surveillance definitions for COVID-19. *European Centre for Disease Prevention and Control*, https://www.ecdc.europa.eu/en/covid-19/surveillance/surveillance-definitions (accessed 17 December 2021).

12. Williamson EJ, Walker AJ, Bhaskaran K, et al. OpenSAFELY: factors associated with COVID-19 death in 17 million patients. *Nature* 2020; 584: 430–436.

13. Lyons RA, Jones KH, John G, et al. The SAIL databank: linking multiple health and social care datasets. *BMC Med Inform Decis Mak* 2009; 9: 3.

14. Simpson CR, Robertson C, Vasileiou E, et al. Early Pandemic Evaluation and Enhanced Surveillance of COVID-19 (EAVE II): protocol for an observational study using linked Scottish national data. *BMJ Open* 2020; 10: e039097.

15. Zhu Y, Matsuyama Y, Ohashi Y, et al. When to conduct probabilistic linkage vs. deterministic linkage? A simulation study. *J Biomed Inform* 2015; 56: 80–86.

16. Fraser LK, Murtagh FEM, Sheldon T, et al. Health of mothers of children with a life-limiting condition: a protocol for comparative cohort study using the Clinical Practice Research Datalink. *BMJ Open* 2020; 10: e034024.

17. Ward JL, Harwood R, Smith C, et al. Risk factors for PICU admission and death among children and young people hospitalized with COVID-19 and PIMS-TS in England during the first pandemic year. *Nat Med* 2021; 1–8.

18. Adeloye D, Katikireddi SV, Woolford L, et al. Uptake, effectiveness and safety of COVID-19 vaccines in children and young people in Scotland: protocol for early pandemic evaluation and enhanced surveillance of COVID-19 (EAVE II). *J Glob Health*.

19. Curtis LA, Burns A. *Unit Costs of Health & Social Care 2020*. PSSRU, University of Kent, https://www.pssru.ac.uk/project-pages/unit-costs/unit-costs-2020/ (2020, accessed 9 December 2021).

For peer review only

20. Swann OV, Pollock L, Holden KA, et al. Comparison of children and young people admitted with SARS-CoV-2 across the UK in the first and second pandemic waves: prospective multicentre observational cohort study. 2021; 2021.09.14.21263567.

21. Green MA, García-Fiñana M, Barr B, et al. Evaluating social and spatial inequalities of large scale rapid lateral flow SARS-CoV-2 antigen testing in COVID-19 management: An observational study of Liverpool, UK (November 2020 to January 2021). *Lancet Reg Health - Eur* 2021; 6: 100107.

22. Jung T, Wickrama K a. S. An Introduction to Latent Class Growth Analysis and Growth Mixture Modeling. *Soc Personal Psychol Compass* 2008; 2: 302–317.

23. Frühwirth-Schnatter S, Pamminger C. Model-based clustering of categorical time series. *Bayesian Anal* 2010; 5: 345–368.

24. Franses PH, Wiemann T. Intertemporal Similarity of Economic Time Series: An Application of Dynamic Time Warping. *Comput Econ* 2020; 56: 59–75.

25. Magnusson K, Skyrud KD, Suren P, et al. Healthcare use in 700 000 children and adolescents for six months after covid-19: before and after register based cohort study. *BMJ* 2022; 376: e066809.

26. People with a positive lateral flow test no longer required to take confirmatory PCR test. *GOV.UK*, https://www.gov.uk/government/news/people-with-a-positive-lateral-flow-test-no-longer-required-to-take-confirmatory-pcr-test (accessed 18 January 2022).

27. Lyons J, Akbari A, Torabi F, et al. Understanding and responding to COVID-19 in Wales: protocol for a privacy-protecting data platform for enhanced epidemiology and evaluation of interventions. *BMJ Open* 2020; 10: e043010.

28. Jones KH, Ford DV, Jones C, et al. A case study of the Secure Anonymous Information Linkage (SAIL) Gateway: a privacy-protecting remote access system for health-related research and evaluation. *J Biomed Inform* 2014; 50: 196–204.

For peer review only

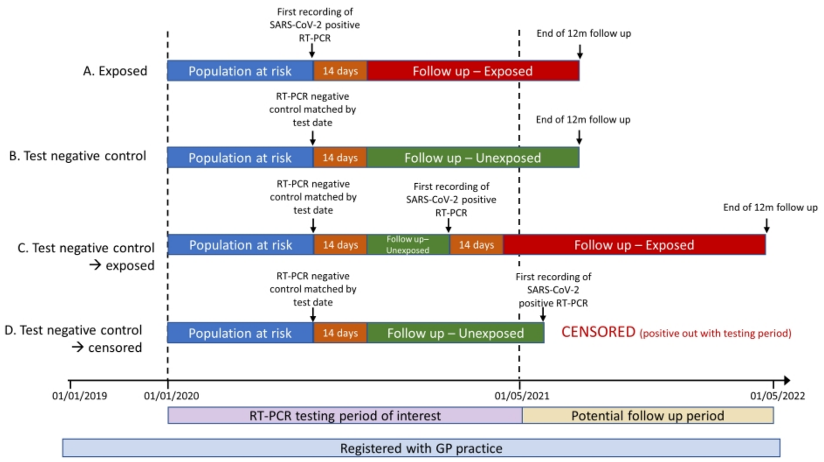

Figure 1. Graphical illustrations of potential study scenarios with test negative controls.

Example A: Positive SARS-CoV-2 RT-PCR case.
Individual A is followed-up from 14 days after SARS-CoV-2 infection for 12 months.

Examples B-D: Test negative controls.
Individual B is matched to an individual with SARS-CoV-2 infection and followed-up from 14 days after matching for 12 months.
Individual C is matched to an individual with SARS-CoV-2 infection and followed-up from 14 days after matching until they are first recorded with SARS-CoV-2 infection themselves during the RT-PCR testing period of interest. At this point they are censored from further follow-up as a test negative comparator and followed-up as an exposed case from 14 days after infection for 12 months with appropriate matches for the date of positive RT-PCR.
Individual D is matched to an individual with SARS-CoV-2 infection and followed-up from 14 days after matching until they are first recorded with SARS-CoV-2 infection themselves. As this occurs after the RT-PCR testing period of interest, they are censored from further follow-up as an unexposed comparator.

338x190mm (300 x 300 DPI)

Studying the Long-term Impact of COVID-19 in Kids (SLICK). Healthcare use and costs in children and young people following community-acquired SARS-CoV-2 infection: protocol for an observational study using linked primary and secondary routinely collected healthcare data from England, Scotland and Wales.

Supplementary Information

***Supplementary Table 1** – Datasets available and Trusted Research Environments*

Country	Trusted Research Environment	Datasets and linkages
Scotland	Scottish National Safe Haven	**SMR00** – Outpatient appointments and attendances<br>**SMR01** – General acute inpatient and day case<br>**SMR04** – Mental health inpatient and day case<br>**SMR06** – Scottish cancer registry<br>**COVID Tests** – Laboratory SARS-CoV-2 tests<br>**Prescribing Information System** – Community prescriptions<br>**Accident and Emergency Datamart**<br>**GP out of hours**<br>**Scottish Ambulance Service**<br>**NHS24 calls**<br>**NRS Deaths**<br>**NRS Infant deaths**<br>**EAVE II** – Scheduled and unscheduled primary care<br>**ISARIC4C/CO-CIN**<br>**COGUK** – SARS-CoV-2 variant<br>**TVMT** – SARS-Cov-2 vaccination data
England	OpenSAFELY	**TPP** - Primary Care<br>**SGSS COVID testing data**<br>**ONS death certificates** – available from 2019-02-01<br>**SUS APCS** (inpatient hospital) – available from 2016-04-01<br>**SUS OPA** (outpatient hospital) – available from 2019-04-01<br>**SUS ECDS** (emergency care) – available from 2017-10-01<br>**ISARIC4C/CO-CIN**
Wales	SAIL	**ConCOV** - Wales Multimorbidity Cohort (WMC) - COVID-19<br>**WLGP** – Primary care<br>**PEDW** – Secondary care (inpatient & day case)<br>**ADDE** – ONS mortality data<br>**CCDS** – Critical care<br>**CDDS** – Consolidate deaths from COVID-19

For peer review only

		**CENW** – Census 2011<br>**CTTP** – COVID-19 test, trace and protect<br>**CVLF** – COVID-19 lateral flow tests<br>**CVSP** – COVID-19 shielded people<br>**CVVD** – COVID-19 vaccines<br>**EDDD** – Emergency department (daily)<br>**EDDS** – Emergency department<br>**ICCD** – intensive care national audit (COVID only admissions)<br>**ICNC** – intensive care national audit<br>**MIDS** – Maternity initial screening and birth<br>**NCCH** – National community child health (maternity, childbirth, etc)<br>**NHSO** – NHS 111, out of hours<br>**OPDW** – Outpatients<br>**OPRD** – Outpatient referrals<br>**PATD** – COVID-19 lab tests<br>**RTTD** – Referral to treatment times<br>**WASD** – Welsh ambulance service<br>**WCSU** – Welsh cancer incidence surveillance unit<br>**WDDS** – Welsh prescription dispensing<br>**WDSD** – Individuals registered with GP, addresses/household information<br>**CRCS** – Children in care or receiving support register<br>**CYFI** – Cystic Fibrosis register<br>**BREC** – Register of all children in Wales with type 1 diabetes<br>**ISARIC4C/CO-CIN**

***Abbreviations: SMR**: Scottish Morbidity Record, **NRS**: National Records of Scotland **EAVE-II:** Early Pandemic Evaluation and Enhanced Surveillance of COVID-19, **ISARIC/CO-CIN**: International Severe Acute Respiratory and emerging Infection Consortium / COVID-19 Clinical Information Network, **COGUK:** COVID-19 Genomics UK Consortium, **TMVT**:Turas Vaccination Management Tool, **NHS**: National Health Service, **TPP:** The Phoenix Partnership (GP group), **SGSS**: Second Generation Surveillance System, **ONS**: Office for National Statistics, **SUS**: Secondary Use Services, **APCS**: Admitted patient care statistics, **OPA**: Outpatient attendances, **ECDS**: Emergency care datasets, **SAIL:** Secure Anonymised Information Linkage, **WLGP**: Welsh Longitudinal General Practice, **PEDW**: Patient Episode Database for Wales, **ADDE**: Annual District Death Extract, **CCDS:** Critical Care Data Source, **CDDS:** COVID-19 Consolidated Deaths, **CENW:** Office of National Statistics Census, **CTTP:** COVID-19 Test, Trace & Protect, **CVLF:** COVID-19 Lateral Flow, **CVSP:** COVID-19 Shielded People, **CVVD:** COVID-19 Vaccine Data, **EDDD:** Emergency Department Dataset Daily, **EDDS:** Emergency Department Dataset, **ICCD:** Intensive Care National Audit & Research Centre (ICNARC) - COVID only admissions, **ICNC:** Intensive Care National Audit & Research Centre (ICNARC), **MIDS:** Maternity Indicators Dataset, **NCCH:** National Community Child Health, **NHSO:** NHS 111 Call data, **OPDW:** Outpatient Dataset for Wales, **OPRD:** Outpatient Referral Dataset, **PATD:** Pathology Data (COVID-19 daily), **RTTD:** Referral to Treatment Times Dataset, **WASD:** Welsh Ambulance Service Dataset, **WCSU:** Welsh Cancer Incidence Surveillance Unit, **WDDS:** Welsh Dispensing Dataset, **WDSD:** Welsh Demographic Service Dataset.*

For peer review only

Supplementary Figure A – Dataflow diagram for Wales

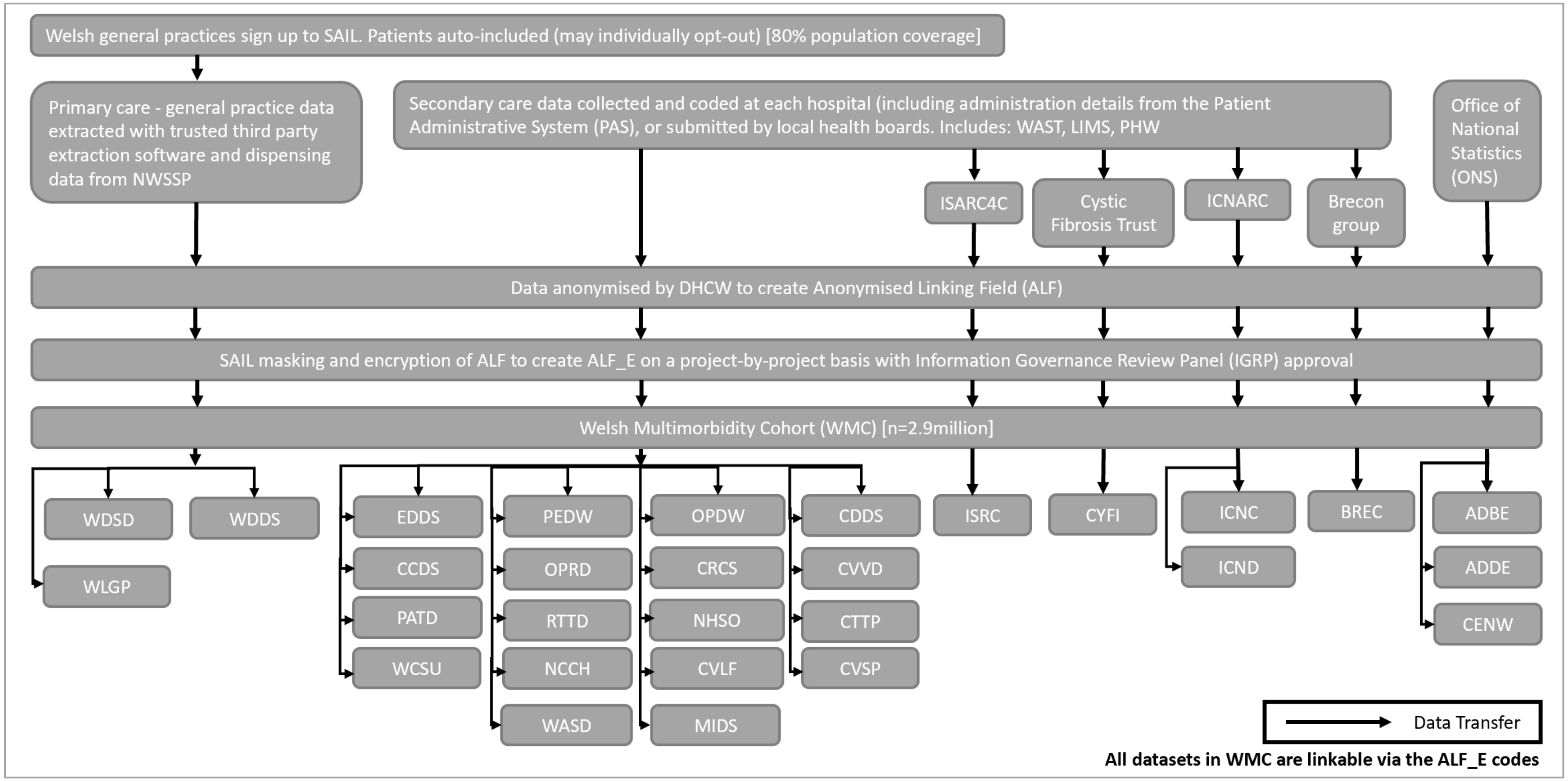

Abbreviations: SAIL: *Secure Anonymised Information Linkage,* ***WLGP****: Welsh Longitudinal General Practice,* ***PEDW****: Patient Episode Database for Wales,* ***ADDE****: Annual District Death Extract,* ***BREC:*** *Brecon Cohort (Children with type 1 diabetes register),* ***CCDS:*** *Critical Care Data Source,* ***CDDS:*** *COVID-19 Consolidated Deaths,* ***CENW:*** *Office of National Statistics Census,* ***CRCS:*** *Children Receiving Care & Support Services,* ***CTTP:*** *COVID-19 Test, Trace & Protect,* ***CVLF:*** *COVID-19 Lateral Flow,* ***CVSP:*** *COVID-19 Shielded People,* ***CVVD:*** *COVID-19 Vaccine Data,* ***CYFI:*** *Cystic Fibrosis Register,* ***EDDS:*** *Emergency Department Dataset,* ***ICCD:*** *Intensive Care National Audit & Research Centre (ICNARC) - COVID only admissions,* ***ICNC:*** *Intensive Care National Audit & Research Centre (ICNARC),* ***ISRC:*** *International Severe Acute Respiratory & Emerging Infection Consortium,* ***ISARIC4C:*** *International Severe Acute Respiratory & Emerging Infection Consortium (Coronavirus Clinical Characterisation Consortium),* ***MIDS:*** *Maternity Indicators Dataset,* ***NCCH:*** *National Community Child Health,* ***NHSO:*** *NHS 111 Call data,* ***OPDW:*** *Outpatient Dataset for Wales,* ***OPRD:*** *Outpatient Referral Dataset,* ***PATD:*** *Pathology Data (COVID-19 daily),* ***RTTD:*** *Referral to Treatment Times Dataset,* ***WASD:*** *Welsh Ambulance Service Dataset,* ***WCSU:*** *Welsh Cancer Incidence Surveillance Unit,* ***WDDS:*** *Welsh Dispensing Dataset,* ***WDSD:*** *Welsh Demographic Service Dataset.*

Supplementary Figure B – Dataflow diagram for Scotland

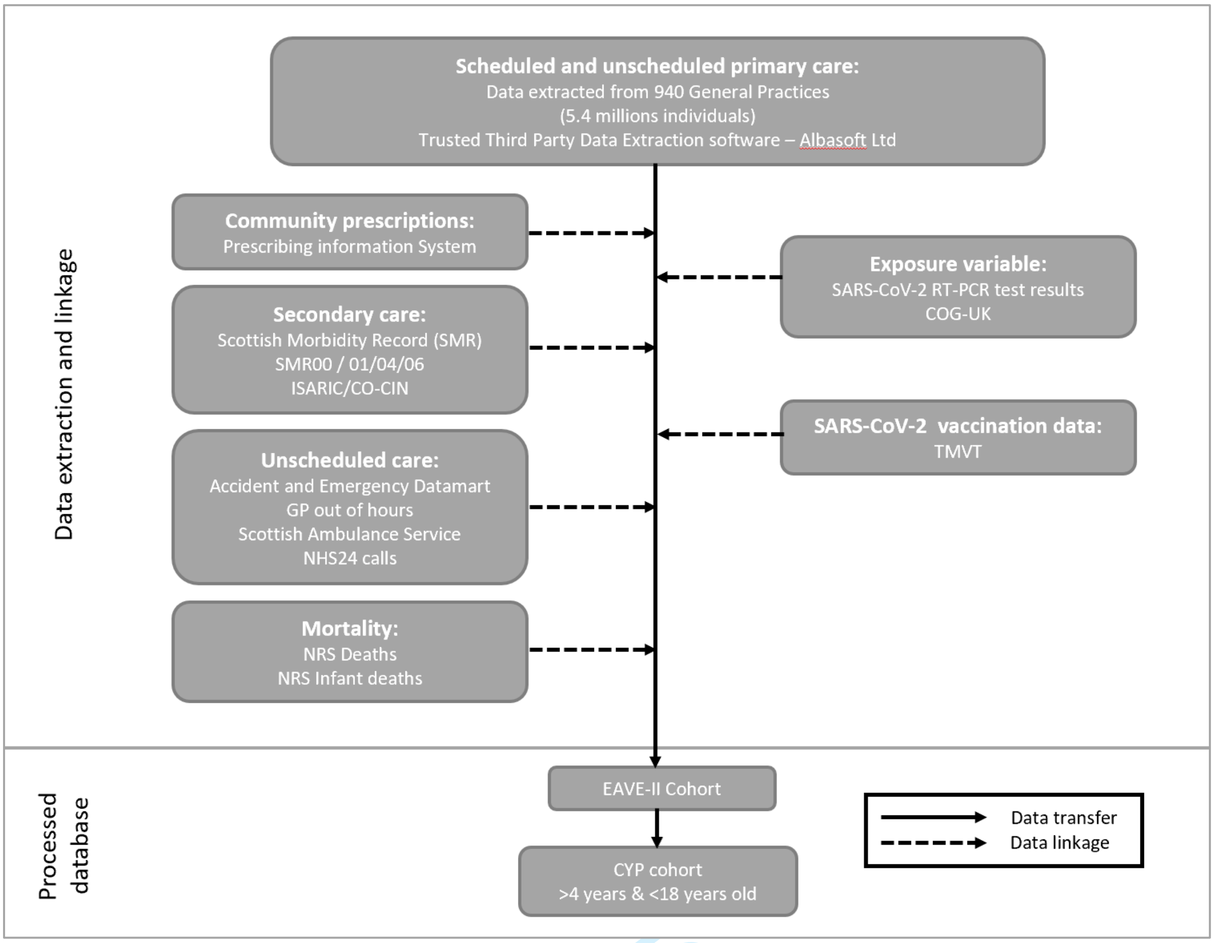

***Abbreviations: ISARIC/CO-CIN**: International Severe Acute Respiratory and emerging Infection Consortium / COVID-19 Clinical Information Network, **GP**: general practice; **NHS**: National Health Service, **NRS**: National Records of Scotland; **RT-PCR**: reverse transcription polymerase chain reaction, **TMVT**: Turas Vaccination Management Tool; **COG-UK:** Centre of Genomics United Kingdom, **CYP**: children and young people*

For peer review only - http://bmjopen.bmj.com/site/about/guidelines.xhtml

Supplementary Figure C – Dataflow diagram for England

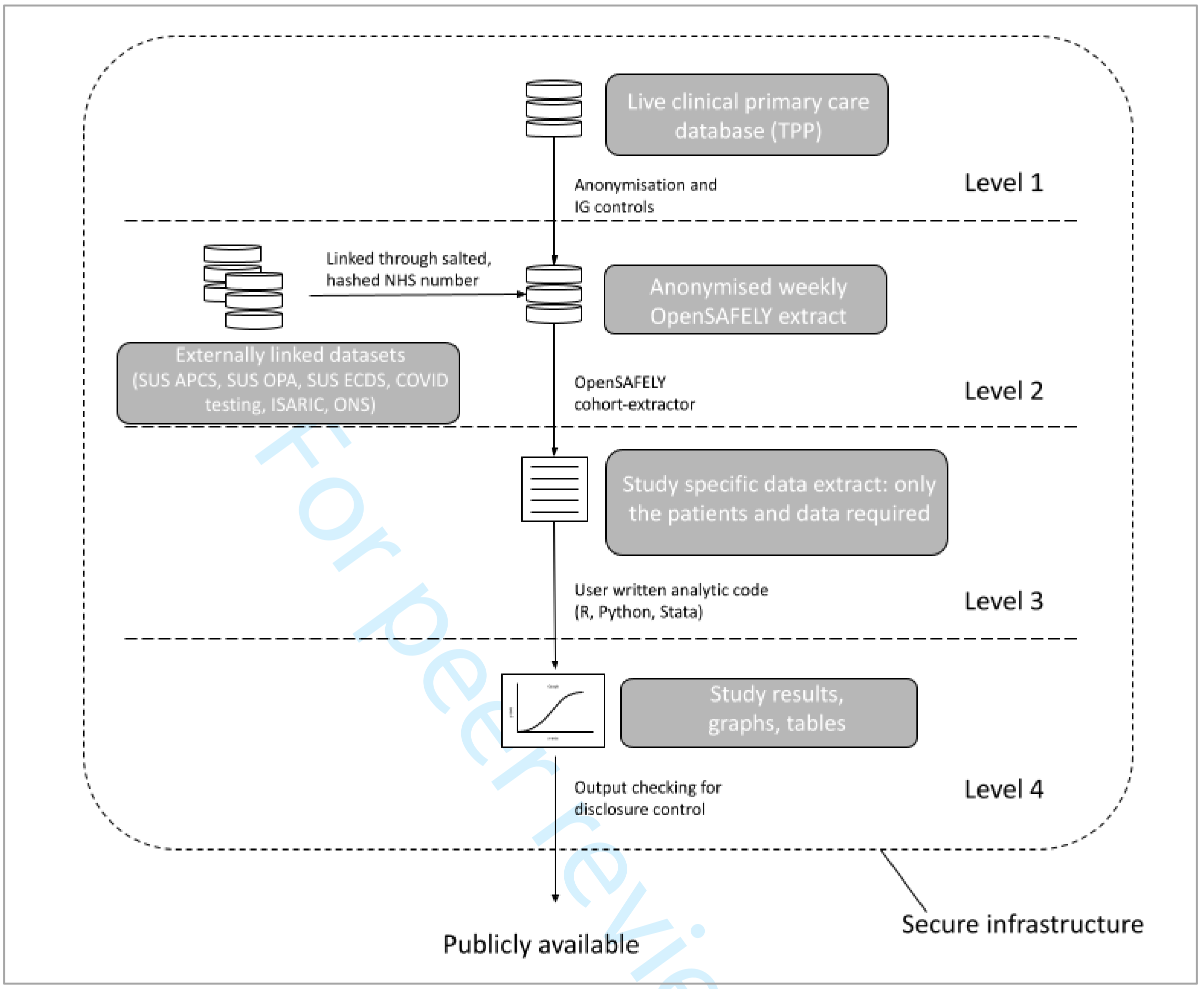

Abbreviations: TPP: *The Phoenix Partnership (GP group),* ***SGSS****: Second Generation Surveillance System,* ***ONS****: Office for National Statistics,* ***SUS****: Secondary Use Services,* ***APCS****: Admitted patient care statistics,* ***OPA****: Outpatient attendances,* ***ECDS****: Emergency care datasets,* ***ONS****: Office for National Statistics.*

For peer review only

For peer review only - http://bmjopen.bmj.com/site/about/guidelines.xhtml

Supplementary Figure D– Directed acyclic graph factors associated with SARS-CoV-2 RT-PCR testing and healthcare use

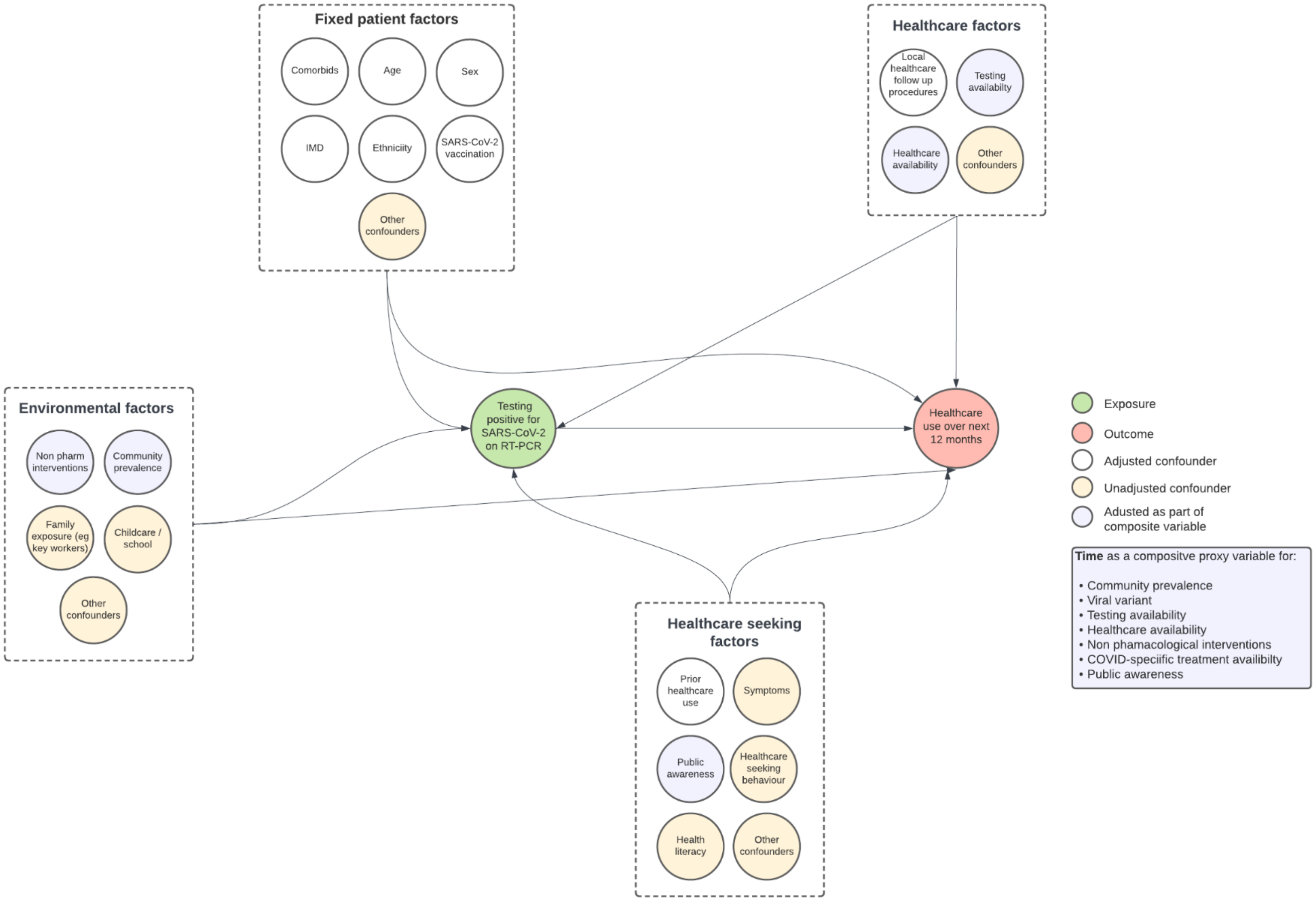

Supplementary Figure E. Graphical illustration of potential study scenarios with population controls (Objective 2 sensitivity analysis)

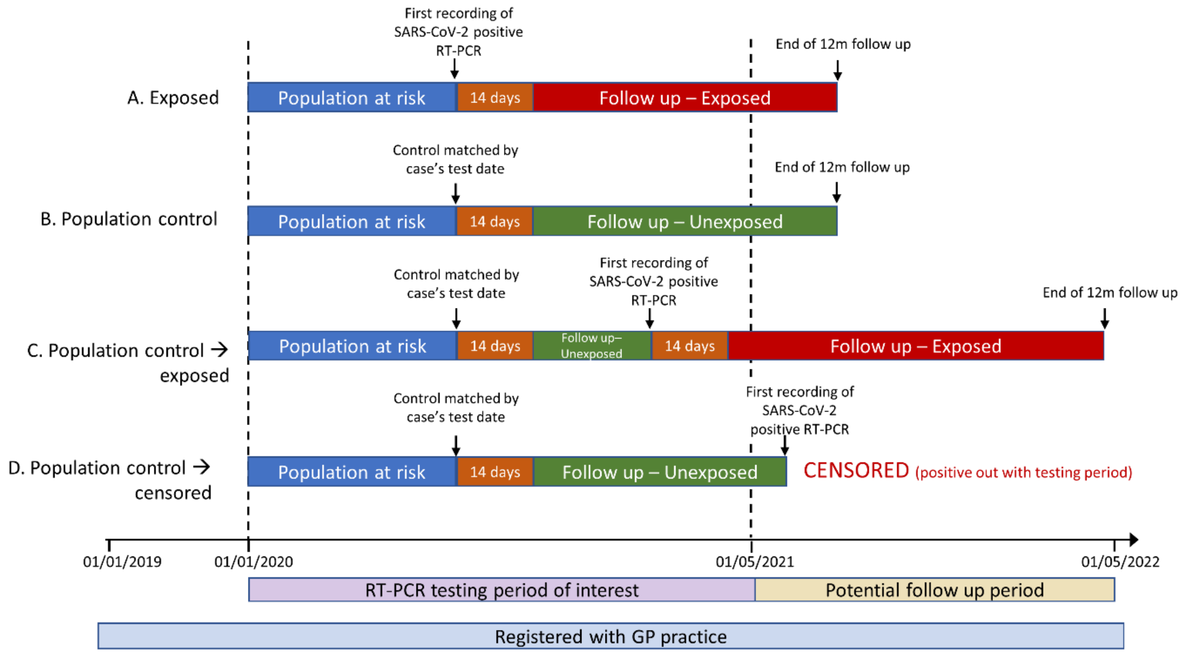

Example A: Positive SARS-CoV-2 RT-PCR case. Individual A is followed-up from 14 days after SARS-CoV-2 infection for 12 months. **Examples B-D: Population controls. Individual B** is matched to an individual with SARS-CoV-2 infection and followed-up from 14 days after matching for 12 months. **Individual C** is matched to an individual with SARS-CoV-2 infection and followed-up from 14 days after matching until they are first recorded with SARS-CoV-2 infection themselves during the RT-PCR testing period of interest. At this point they are censored from further follow-up as a test negative comparator and followed-up as an exposed case from 14 days after infection for 12 months with appropriate matches for the date of positive RT-PCR. **Individual D** is matched to an individual with SARS-CoV-2 infection and followed-up from 14 days after matching until they are first recorded with SARS-CoV-2 infection themselves. As this occurs after the RT-PCR testing period of interest, they are censored from further follow-up as an unexposed comparator.

For peer review only

For peer review only - http://bmjopen.bmj.com/site/about/guidelines.xhtml

Supplementary Figure F – Logo for SLICK study

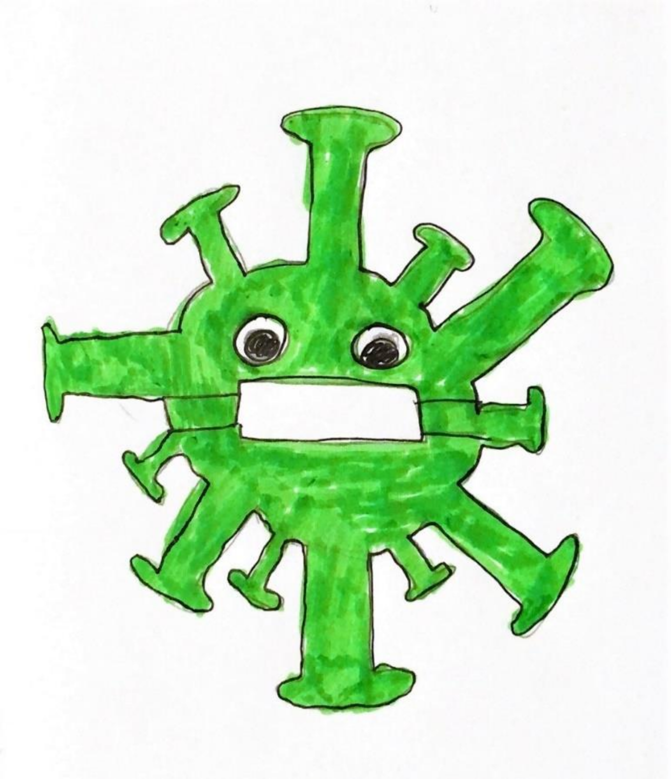

Designed by Georgia Langley, aged 11

For peer review only - http://bmjopen.bmj.com/site/about/guidelines.xhtml

BMJ Open

Studying the Long-term Impact of Covid in Kids (SLICK). Healthcare use and costs in children and young people following community-acquired SARS-CoV-2 infection: protocol for an observational study using linked primary and secondary routinely collected healthcare data from England, Scotland and Wales.

Journal:	*BMJ Open*
Manuscript ID	bmjopen-2022-063271.R1
Article Type:	Protocol
Date Submitted by the Author:	20-Sep-2022
Complete List of Authors:	Swann, Olivia; The University of Edinburgh Usher Institute of Population Health Sciences and Informatics, Centre for Medical Informatics; The University of Edinburgh Department of Child Life and Health,<br>Lone, Nazir; The University of Edinburgh Usher Institute of Population Health Sciences and Informatics, Centre for Population Health Sciences<br>Harrison, Ewen; The University of Edinburgh Usher Institute of Population Health Sciences and Informatics, Centre for Medical Informatics<br>Tomlinson, Laurie; London School of Hygiene and Tropical Medicine Department of Non-communicable Disease Epidemiology<br>Walker, Alex; University of Oxford Nuffield Department of Primary Care Health Sciences, The DataLab<br>Seaborne, Michael; Swansea University, Centre for Population Health<br>Pollock, Louisa; University of Glasgow Department of Child Health<br>Farrell, James; The University of Edinburgh Usher Institute of Population Health Sciences and Informatics, Centre for Medical Informatics<br>Hall, Peter; The University of Edinburgh MRC Institute of Genetics and Molecular Medicine, Institute of Cancer and Genetics<br>Seth, Sohan; The University of Edinburgh School of Informatics<br>Williams, Thomas; The University of Edinburgh Department of Child Life and Health,<br>Preston, Jenny; University of Liverpool Faculty of Humanities and Social Sciences<br>Ainsworth, Jennifer; University of Liverpool Faculty of Humanities and Social Sciences<br>Semple, Freya; University of Glasgow School of Medicine Dentistry and Nursing<br>Baillie, J; Roslin Institute, Division of Genetics and Genomics<br>Katikireddi, Srinivasa; University of Glasgow, MRC/CSO Social & Public Health Sciences Unit<br>Akbari, Ashley; Swansea University Medical School; Swansea University Medical School, Health Data Research UK<br>Lyons, Ronan; Swansea University Medical School<br>Simpson, Colin; The University of Edinburgh, Usher Institute of Population Health Sciences and Informatics

	Semple, Malcolm G; NIHR Health Protection Research Unit in Emerging and Zoonotic Infections at University of Liverpool; Alder Hey Children's Hospital, Respiratory Paediatrics<br>Goldacre, Ben; University of Oxford Nuffield Department of Primary Care Health Sciences, The DataLab<br>Brophy, Sinead; Swansea University Medical School, Health Data Research UK<br>Sheikh, Aziz; The University of Edinburgh Usher Institute of Population Health Sciences and Informatics<br>Docherty, Annemarie; The University of Edinburgh Usher Institute of Population Health Sciences and Informatics, Centre for Medical Informatics; University of Edinburgh, Anaesthesia and Critical Care
**Primary Subject Heading**:	Infectious diseases
Secondary Subject Heading:	Epidemiology, Paediatrics
Keywords:	COVID-19, Epidemiology < INFECTIOUS DISEASES, Paediatric infectious disease & immunisation < PAEDIATRICS, HEALTH ECONOMICS

SCHOLARONE™
Manuscripts

Studying the Long-term Impact of COVID-19 in Kids (SLICK). Healthcare use and costs in children and young people following community-acquired SARS-CoV-2 infection: protocol for an observational study using linked primary and secondary routinely collected healthcare data from England, Scotland and Wales.

For peer review only

Author list and affiliations

Olivia V. Swann [1, 2 *], Nazir Lone [3], Ewen M. Harrison [1], Laurie A. Tomlinson [4], Alex J. Walker [5], Michael Seaborne [6], Louisa Pollock [7], James Farrell [1], Peter S Hall [8], Sohan Seth [9], Thomas C. Williams [2], Jennifer Preston [10], J. Samantha Ainsworth [10], Freya F. Semple [11], J. Kenneth Baillie [12], Srinivasa V Katikireddi [13], Ashley Akbari [14], Ronan A Lyons [14], Colin R Simpson [3, 15], Malcolm G. Semple [16], Ben Goldacre [5], Sinead Brophy [17], Aziz Sheikh [3] and Annemarie B. Docherty [1]

* *Corresponding author*: Dr Olivia Swann, PhD, Clinical Lecturer in Paediatric Infectious Diseases, Centre for Medical Informatics, Usher Institute, University of Edinburgh, Edinburgh, UK.

Email: Olivia.Swann@ed.ac.uk

Affiliations

1. Centre for Medical Informatics, Usher Institute, University of Edinburgh, Edinburgh, UK
2. Department of Child Life and Health, University of Edinburgh, Edinburgh, UK
3. Usher Institute, University of Edinburgh, Edinburgh, UK
4. Department of Non-Communicable Disease Epidemiology, London School of Hygiene and Tropical Medicine, London, United Kingdom.

5. The DataLab, Nuffield Department of Primary Care Health Sciences, University of Oxford, Oxford, UK
6. Centre for Population Health, Swansea University, Swansea, UK
7. Child Health, School of Medicine, Dentistry and Nursing, University of Glasgow, Glasgow, UK
8. Institute of Cancer and Genetics, University of Edinburgh, Edinburgh, UK
9. School of Informatics, University of Edinburgh, Edinburgh, UK
10. Faculty of Humanities & Social Sciences, University of Liverpool
11. School of Medicine, Dentistry and Nursing, University of Glasgow, Glasgow, UK
12. Roslin Institute, University of Edinburgh, Edinburgh, UK
13. MRC/CSO Social & Public Health Sciences Unit, University of Glasgow, Glasgow, UK
14. Swansea University Medical School, Swansea University, Swansea, UK
15. School of Health, Faculty of Health, Victoria University of Wellington, Wellington, New Zealand
16. NIHR Health Protection Research Unit in Emerging and Zoonotic Infections and Institute of Infection, Veterinary and Ecological Sciences, Faculty of Health and Life Sciences, University of Liverpool, Liverpool, UK
17. Health Data Research UK, Swansea University Medical School, Swansea University, Swansea, UK.

For peer review only

Abstract

Introduction

Severe acute respiratory syndrome coronavirus 2 (SARS-CoV-2) infection rarely causes hospitalisation in children and young people (CYP), but mild or asymptomatic infections are common. Persistent symptoms following infection have been reported in CYP but subsequent healthcare use is unclear. We aim to describe healthcare use in CYP following community-acquired SARS-CoV-2 infection and identify those at risk of ongoing healthcare needs.

Methods and analysis

We will use anonymised individual-level, population-scale national data linking demographics, comorbidities, primary and secondary care use and mortality between 01/01/2019-01/05/2022. SARS-CoV-2 test data will be linked from 01/01/20-01/05/2022. Analyses will use Trusted Research Environments: OpenSAFELY in England, Secure Anonymised Information Linkage (SAIL Databank) in Wales and Early Pandemic Evaluation and Enhanced Surveillance of COVID-19 (EAVE-II) in Scotland. CYP aged ≥4 and <18 years who underwent SARS-CoV-2 reverse transcription polymerase chain reaction (RT-PCR) testing between 01/01/20 and 01/05/21 and those untested CYP will be examined.

The primary outcome measure is cumulative healthcare cost over 12 months following SARS-CoV-2 testing, stratified into primary or secondary care, and physical or mental healthcare. We will estimate the burden of healthcare use attributable to SARS-CoV-2 infections in the 12 months after testing using a matched cohort study of RT-PCR positive, negative or untested CYP matched on testing date, with adjustment for confounders. We will identify factors associated with higher healthcare needs in the 12 months following SARS-CoV-2 infection using an unmatched cohort of RT-PCR positive CYP. Multivariable logistic regression and machine learning approaches will identify risk factors for high healthcare use and characterise patterns of healthcare use post infection.

Ethics and dissemination

This study was approved by the South-Central Oxford C Health Research Authority Ethics Committee (13/SC/0149). Findings will be pre-printed and published in peer-reviewed

For peer review only

journals. Analysis code and code-lists will be available through public GitHub repositories and OpenCodelists with meta-data via HDR-UK Innovation Gateway.

For peer review only

Article Summary

Strengths:

1. Objective, direct examination of clinician-recorded healthcare use by CYP post SARS-CoV-2 infection.
2. Population-wide coverage of all children and young people (CYP) <18 years in Scotland and Wales and approximately 4.8 million CYP in England.

Limitations:

1. Lack of access to SARS-CoV-2 lateral flow testing (rapid antigen testing) results may result in misattribution of SARS-CoV-2 status in patients when reverse transcription polymerase chain reaction (RT-PCR) testing was not performed.
2. Access to health services is presumed to be available for anyone who needed it, but this may have been reduced by local healthcare policies and patient health-seeking behaviour at different points during the pandemic.
3. Owing to the time needed for 12 months of follow up, this study will focus on healthcare use after infection with wildtype and Alpha variants of SARS-CoV-2, which may differ from Delta and Omicron.

Introduction

Severe acute respiratory syndrome coronavirus 2 (SARS-CoV-2) causes the disease COVID-19, with adults being more severely affected than children throughout the pandemic [1]. While hospitalisation with SARS-CoV-2 is rare in children and young people (CYP) [2], infection is common, with up to 70% (95% CI 68-71) of 5-14 year olds estimated to have been infected with SARS-CoV-2 in the UK by December 2021 [3]. Whilst research on COVID-19 in CYP has focused on index hospitalisations and deaths, this acute view means we have not established what the additional healthcare needs are for the majority of CYP after mild or asymptomatic SARS-CoV-2 infection. There is also little information on the changes to healthcare use in children with co-morbidities who may be at risk of exacerbations (for example asthma). The large numbers of CYP infected with SARS-CoV-2 in the UK means that even a small increase in healthcare use in this population could substantially impact on

For peer review only

healthcare services. Being asymptomatic with initial infection does not guarantee against developing subsequent illness from SARS-CoV-2, for example CYP who are asymptomatic with their initial SARS-CoV-2 infection can develop Multisystem Inflammatory Syndrome in Children (MIS-C) two to eight weeks later [4]. Whilst this complication is extremely rare (approximately 3 cases per 10,000 infections [5]), it underlines the need to include CYP who are initially asymptomatic from SARS-CoV-2 infection when examining subsequent healthcare use.

A wide variety of persistent symptoms have been reported in CYP following SARS-CoV-2 infection with studies varying in design and quality (reviewed in [6]). Most reports have used a questionnaire or clinic-based approach to symptom reporting, often after hospitalisation with COVID-19 or in patients self-identifying as having Long-COVID, introducing significant potential sources of bias.

Data on long term healthcare use following -SARS-CoV-2 infection is beginning to emerge, although most studies have focused on adults rather than CYP. One large study of American adults (n= 5,064,270)reported an increase in outpatient healthcare use in the six months following SARS-CoV-2 infection (hazard ratio of 1.20 (1.19–1.21)[7]. Another American study (*n*=250,514) found COVID-19 diagnosis was associated with an additional 0.7269 (95% CI, 0.7088 to 0.7449) monthly healthcare visits (combined inpatient and outpatient visits excluding respiratory healthcare contacts) in the six months after diagnosis [8].This study did include some CYP (*n* not given) and reported that healthcare use post-COVID-19 diagnosis increased slightly from two to five months after diagnosis for those ≤17 years old, but returned to pre-diagnosis baseline levels by six months.

One Norwegian study examined healthcare use in CYP aged 1-19 years (n=706,885) for six months from SARS-CoV-2 testing and reported an increase in primary healthcare use for all ages during the first one to four weeks following a positive test compared with CYP who tested negative [9]. These presentations were predominantly respiratory. This increase in healthcare use was more sustained in younger CYP, while those aged 16-19 years retuned to baseline healthcare use by five to eight weeks. The study did not find any increase in use of specialist care for any age group.

For peer review only

No studies have yet examined healthcare use in CYP in the United Kingdom (UK) following SARS-CoV-2. Using routinely collected anonymised electronic health record (EHR) data at an individual-level, population-scale matched by SARS-CoV-2 RT-PCR status to examine healthcare use after SARS-CoV-2 infection in CYP offers an alternative method to questionnaire or clinic-based symptom reporting after SARS-CoV-2..In addition to traditional epidemiological approaches, machine learning methods are also proving increasingly important in the analysis of large routinely collected healthcare datasets in SARS-CoV-2 [10]. Using machine learning to identify clusters of patients with similar healthcare trajectories provides a complementary approach to traditional epidemiology o identify patients at risk of high healthcare use post infection. A combination of approaches would establish the long-term healthcare use attributable to SARS-CoV-2 in CYP, which is essential both for tailoring individual care for patients at risk of high healthcare use post infection and informing health service and vaccination planning.

Aims

We aim to establish the patterns and burden of healthcare use in CYP attributable to community-acquired SARS-CoV-2 infection and identify those CYP at risk of high or ongoing healthcare needs in England, Scotland and Wales.

Objectives

We will:

1. Describe the background healthcare use in CYP before and during the pandemic.
2. Compare healthcare use in CYP in the 12 months after testing positive, negative or not being tested for SARS-CoV-2 by RT-PCR to estimate burden of healthcare use attributable to SARS-CoV-2.
3. Identify factors associated with higher healthcare use (including having co-morbidities) in the 12 months following SARS-CoV-2 infection.

For peer review only

Methods

Study period

The period covered by the study will span 01/01/2019 to 01/05/22 and focus on SARS-CoV-2 infections until 01/05/21. This study period was chosen to provide 12 months of follow up data for CYP infected to the end of the second wave of SARS-CoV-2 in the UK (end of April 2021 [11]) as well as those testing negative or not tested. Inclusion of the period from 01/01/19 to 01/01/20 will also provide at least a year of data on pre-pandemic data on healthcare use for each CYP.

Study design

The study will comprise three main approaches; a descriptive graphical analysis addressing Objective 1 (background healthcare use before and during the pandemic), a matched cohort study addressing Objective 2 (estimating healthcare use post SARS-CoV-2 infection) and an unmatched cohort study addressing Objective 3 (identifying factors associated with higher healthcare needs post SARS-CoV-2 infection).

Study Population

The study population will vary with objective:

Inclusion criteria (all objectives):

- Registered with a General Practitioner (GP) in Scotland (includes all general practices), Wales or England (The Phoenix Partnership (TPP) a group of GP practices with a unified electronic patient-record system covering approximately 34% of practices in England [12])

Exclusion criteria (all objectives):

- Positive index SARS-CoV-2 RT-PCR test performed after 7 days in hospital (to exclude nosocomial infections [13])
- CYP with discrepant SARS-CoV-2 RT-PCR results on the same date

Objective 1- Objective-specific inclusion criteria

- Age ≥4 years and <18 years on 01/01/19 (pre-pandemic period)
- Age ≥4 years and <18 years on 01/01/20 (pandemic period – pandemic year 1)
- Age ≥4 years and <18 years on 01/01/21 (pandemic period – pandemic year 2)

For peer review only

Objective 2 - Objective-specific inclusion criteria

- Underwent SARS-CoV-2 PCR testing (or untested but matched to CYP who had been tested) between 01/01/20 and 01/05/21
- Age ≥4 and <18 years on date of testing / matching
- At least 12 months of healthcare data available both before and after SARS-CoV-2 PCR test / date of matching if not tested
- No previous positive SARS-CoV-2 PCR test recorded

Objective 3 - Objective-specific inclusion criteria

- Positive SARS-CoV-2 RT-PCR test between 01/01/20 and 01/05/21
- Age ≥4 and <18 years on date of testing
- At least 12 months of healthcare data available both before and after SARS-CoV-2 PCR test
- No previous positive SARS-CoV-2 PCR test recorded

Data sources and validation

Data will be held securely and analyses conducted within nation-specific Trusted Research Environments (TREs): OpenSAFELY in England [14], Secure Anonymised Information Linkage (SAIL Databank [15]) in Wales and the Early Pandemic Evaluation and Enhanced Surveillance of COVID-19 (EAVE-II) platform [16] within Public Health Scotland in Scotland. TREs provide secure computing environments which hold data remotely and enable access for analysis without the data itself ever leaving the secure site.

OpenSAFELY is a secure, transparent, open-source software platform for analysis of electronic health records data allowing detailed analysis of pseudonymised primary care patient records in England. Other datasets are linked within the same environment using a matching pseudonym derived from the National Health Service (NHS) number.

SAIL Databank brings together electronically-held, person-based, routinely-collected demographic and clinical data across Wales for the purpose of conducting and supporting health-related research, which are pseudonymised using Anonymous Linking Fields.

For peer review only

EAVE-II is a national linked dataset of patient-level primary care data, out-of-hours, hospitalisation, mortality and laboratory data across Scotland. Data is held securely and analysed in the Public Health Scotland TRE.

Deterministic and probabilistic linking of datasets will be carried out via Community Health Index (CHI) number in Scotland and by NHS number in England and Wales. NHS and CHI numbers are unique identifiers used in all health-care contacts across the NHS [17]. Datasets contributing to each country's final database are described in *Supplementary Table 1* with data flow diagrams in *Supplementary Figures A-C*. In addition to the study period outlined, data from birth will also be examined to identify comorbidities, including common chronic childhood conditions [18]. In the event of missing data, these will be supplemented by information for that CYP in linked datasets. All variables will be checked for patterns of missingness and implausible values and a log maintained for reasons where records are excluded from analysis. In cases where an analysis variable has high levels of missingness, alternative variables which are closely related may be considered as a proxy for these missing data. Depending on the cause ascertained for missing variables, we will consider imputation.

As PIMS-TS is a new disease, ICD-10 coding was not introduced until November 2020. Admission will be considered due to PIMS-TS if occurring between 01/02/20 – 01/11/20 and coded as Kawasaki disease, toxic shock syndrome or systemic inflammatory response (proxies for PIMS-TS) or if admitted after 01/11/20 and coded as PIMS-TS [19]. National PIMS-TS databases (available in Scotland and Wales) will be used for sensitivity analyses. The major data sources for each variable are detailed in *Table 1* (adapted from [20]).

For peer review only

Table 1. Groupings of variables by source

	Variable	Data source		
		England (OpenSAFELY)	Scotland (EAVE-II)	Wales (SAIL)
**Demographics**	Sex	TPP	EAVE-II	WDSD/WLGP
	Age	TPP	EAVE-II	WDSD/WLGP
	Ethnicity	TPP	EAVE-II	CENW/NCCH
**Socio-economic**	IMD	TPP	EAVE-II	WDSD
**Place of residence**	Health board / STP, urban rural index	TPP	EAVE-II	WDSD
**Accommodation type**	Private or social housing	*NA*	EAVE-II	CENW
**Comorbidities**	Chronic childhood conditions	TPP, SUS APCS, SUS OPA, ISARIC	EAVE-II, SMR00/01/04/06, ISARIC	CYFI, BREC, WCSU, WLGP, PEDW
	SARS-CoV-2 shielding list	TPP	EAVE-II	CVSP
**SARS-CoV-2 vaccination**	Vaccine (type, date)	TPP	TVMT	CVVD
**Laboratory tests**	RT-PCR SARS-CoV-2 test (date and result)	SGSS	COVID-testing	PATD
	Viral variant	SGSS	COG UK	CVSD/PATD
**Secondary care**	ED contact	SUS ECDS	A+E Datamart	EDDD/EDDS
	Outpatient clinic contact	SUS OPS	SMR00	OPDW
	Hospital admission	SUS APCS	SMR01/04	PEDW
	Admission ICD-10 code	SUS APCS	SMR	PEDW
	Level of care	SUS / ISARIC	SMR / ISARIC	PEDW/CCDS
	Length of stay	SUS / ISARIC	SMR / ISARIC	PEDW/CCDS
	PIMS-TS	SUS / ISARIC	SMR / ISARIC	PEDW/CCDS
**Primary care**	In-hours contact	TPP	EAVE-II	WLGP

For peer review only

	Community prescriptions	TPP	EAVE-II / PIS	WDDS
**Unscheduled care**	NHS 111 contact<br>Ambulance contact<br><br>GP out of hours contact	*NA*<br>*NA*<br><br>*NA*	NHS 24<br>SAS<br><br>GP OOH	NHSO<br>WASD/NHSO<br>NHSO
**Mortality**	Death (all cause, COVID-19 main cause or <28 days of positive SARS-CoV-2 RT-PCR)	ONS deaths	NRS deaths	ONS deaths / ADDE
**Symptoms**	Presenting symptoms in CYP admitted with SARS-CoV-2	ISARIC (subset only)	ISARIC (subset only)	ISARIC (subset only)

Abbreviations: **EAVE-II=**Early Pandemic Evaluation and Enhanced Surveillance of COVID-19; **SAIL**: Secure Anonymised Information Linkage; **IMD**=Index of Multiple Deprivation; **STP**=Sustainability and Transformation Partnership (STP, geographical areas configured for regional reorganisation in England), **ED**= Emergency Department; **ICD-10**=International Classification of Diseases 10th Revision; **NHS**=National Health Service; **GP**=general practice; **PIMS-TS**= Paediatric multisystem inflammatory syndrome temporally associated with COVID-19; **TPP**=The Phoenix Partnership (GP group); **SUS**=Secondary Use Services; **APCS**=Admitted patient care statistics; **OPA**=Outpatient attendances; **ECDS**=Emergency care datasets; **SGSS**=Second Generation Surveillance System; **ISARIC** =International Severe Acute Respiratory and emerging Infection Consortium / COVID-19 Clinical Information Network; **ONS**: Office for National Statistics; **SMR**=Scottish Morbidity Record; **TMVT**=Turas Vaccination Management Tool; **COG-UK**=Centre of Genomics United Kingdom; **PIS**= Prescribing Information System; **SAS**=Scottish Ambulance Service; **OOH**=Out Of Hours; **NRS**=National Records of Scotland; **WLGP**=Welsh Longitudinal General Practice; **PEDW**=Patient Episode Database for Wales; **ADDE**=Annual District Death Extract; **CCDS**=Critical Care Data Source; **CDDS**=COVID-19 Consolidated Deaths; **CENW**=Office of National Statistics Census; **CTTP**=COVID-19 Test Trace & Protect; **CVLF**=COVID-19 Lateral Flow; **CVSP**=COVID-19 Shielded People; **CVVD**=COVID-19 Vaccine Data; **EDDD**=Emergency Department Dataset Daily; **EDDS**=Emergency Department Dataset; **ICCD**=Intensive Care National Audit & Research Centre (ICNARC)-COVID only admissions; **ICNC**=Intensive Care National Audit & Research Centre (ICNARC); **MIDS**=Maternity Indicators Dataset; **NCCH**=National Community Child Health; **NHSO**=NHS 111 Call data; **OPDW**=Outpatient Dataset for Wales; **OPRD**=Outpatient Referral Dataset; **PATD**=Pathology Data (COVID-19 daily); **RTTD**=Referral to Treatment Times Dataset; **WASD**=Welsh Ambulance Service Dataset; **WCSU**=Welsh Cancer Incidence Surveillance Unit; **WDDS**=Welsh Dispensing Dataset; **WDSD**=Welsh Demographic Service Dataset. **NA**=Not available.

Exposure

The exposure of interest is diagnosis of SARS-CoV-2 infection, defined as a positive RT-PCR test result. The date of exposure is defined as the date of the positive RT-PCR test result.

For peer review only

Outcomes

The primary outcome measure will be cumulative NHS healthcare costs over the 12 months following SARS-CoV-2 testing. This will provide an overarching measure that is reflective of healthcare resource use, which is expressed on a monetary scale that is common between the three nations and common to all types of activity. Activity will only contribute to the primary outcome measure if it is quantifiable from data in all three nations. Healthcare costs will be broken down into budget-holder perspectives; secondary care (critical care/inpatient/outpatient/A&E) and primary care (face-to face or telephone in-hours primary care activity). A sensitivity analysis of unscheduled care (e.g. NHS 24, ambulance, GP OOH) will be undertaken for the nations where this data is available (Scotland and Wales). To ensure comparability, unit costs will be assigned from a common country (England) using Personal Social Services Research Unit costs with a common base year [21].

Secondary outcomes will constitute units of healthcare activity, quantifiable as counts over time or rates, that can be quantified to a common definition between the three nations, e.g. inpatient episodes by specialty or primary care appointments. Both primary and secondary outcomes will be stratified into predominantly physical or mental healthcare based on the primary reason for admission / attendance. The reason for healthcare use will also be further explored (e.g. by body system / healthcare speciality).

Statistical analyses

Analyses will be replicated across the three nations in each respective TRE.

Objective 1

Describe the background healthcare use in CYP before and during the pandemic.

Significant, dynamic changes in both healthcare access and healthcare-seeking behaviour have occurred across the course of the pandemic to date. As such, exploration of background healthcare use in CYP before and during the pandemic will help contextualise subsequent analyses. A descriptive, graphical analysis will be undertaken. Healthcare use (represented as cost) will be plotted for the period of 01/01/19 to 01/05/22 for all CYP. These data will be stratified by variables including age, sex, nation of residence, type of healthcare

For peer review only

(primary or secondary care) and RT-PCR status (RT-PCR positive, RT-PCR negative and never tested). Reasons for healthcare visits will also be explored.

Objective 2

Compare healthcare use in CYP in the 12 months after testing positive, negative or not being tested for SARS-CoV-2 by RT-PCR to estimate the burden of healthcare use attributable to SARS-CoV-2.

This analysis will focus on estimating the burden of CYP healthcare use which is attributable to SARS-CoV-2 infection in the 12 months after infection, whereas individual factors associated with healthcare use after infection will be explored in Objective 3. As well as total healthcare use in the 12 months following SARS-CoV-2 infection, we will also break this objective into 0-3, 3-6, 6-9 and 9-12 month brackets to examine how healthcare use changes across time.

A prospective matched cohort study will be undertaken. Matching will be undertaken for date of RT-PCR test with iterative widening bands as necessary. This will account for availability of testing, access to healthcare, variation in incidence rates, emergence of viral variants, changes in SARS-CoV-2 treatment and systematically different characteristics in the tested population (compared to the untested population) as the pandemic progressed (*Supplementary Figure D)*. Ten RT-PCR test negative non-hospitalised control CYP will be matched without replacement for every RT-PCR positive case.

Stabilised inverse probability weights will be used to adjust for known confounder imbalance between cases and controls. The following variables will be explored: age, sex, SARS-CoV-2 vaccination status at the time of index RT-PCR test (considered vaccinated if ≥3 weeks since first dose), geographical region (health board / Sustainability and Transformation Partnership (STP)) to account for regional differences in RT-PCR testing and availability of healthcare, previous healthcare contact (primary or secondary), chronic conditions, number of previous SARS-CoV-2 tests, socioeconomic status (quintiles of relevant national deprivation measure: Scottish Index of Multiple Deprivation (SIMD), Welsh Index of Multiple Deprivation (WIMD) and Lower layer Super Output Area (LSOA)) and urban-rural index.

For peer review only

Factors are associated with being brought for RT-PCR testing (e.g. public awareness and testing availability) may be different from those of exposure to SARS-CoV-2. A directed acyclic graph of factors associated with SARS-CoV-2 RT-PCR testing and healthcare use to consider in model building is shown in *Supplementary Figure D.*

In contrast to adults, the median hospital length of stay due to SARS-CoV-2 in CYP is short, previously reported in the UK as 2 days (IQR 1-4) [22]. As such, follow up will start 14 days after testing positive for SARS-CoV-2 on RT-PCR which will enable us to look back and further stratify the exposure by SARS-CoV-2 severity (i.e. community care, hospitalisation or critical care).

CYP in the control group may subsequently test positive for SARS-CoV-2 by RT-PCR. If this occurs during the RT-PCR testing period of interest (01/01/20 - 01/05/21) they will become a case and follow-up commenced for 12 months (with appropriate matches for the date of the positive RT-PCR). If the control tests positive after 01/05/21 (i.e. after the RT-PCR testing period of interest), they will be censored and will not become a case. A graphical illustration of the potential CYP paths for this analysis is shown in *Figure 1.*

For peer review only

As CYP who are brought for RT-PCR testing are systematically different to those who are not brought [23], a sensitivity analysis will be undertaken to compare the RT-PCR positive cohort against the population of CYP who have never tested positive (i.e. both RT-PCR negative and untested CYP), hereafter "population controls." RT-PCR positive CYP will be matched to ten population controls who were not hospitalised on the date of their matched case's RT-PCR [7]. Confounding will then be minimised as described above. A graphical illustration of the potential CYP paths for this analysis is shown in *Supplementary Figure E.*

The proportion of CYP with SARS-CoV-2 infection but without a positive RT-PCR (e.g. tested by lateral flow (rapid antigen test) or untested asymptomatic cases) has increased across the pandemic [3]. As such, we will conduct quantitative bias analyses for unmeasured confounding using different estimates of undetected SARS-CoV-2 infection across the study period.

Objective 3

Identify factors associated with higher healthcare use (including having co-morbidities) in the 12 months following SARS-CoV-2 infection.

Both regression and machine learning approaches will be undertaken to examine healthcare costs in the SARS-CoV-2 RT-PCR positive cohort. A multivariable regression model will be constructed with covariates including demographics (age, sex, socioeconomic status, urban-rural Index and health board / STP, pre-existing health status (chronic comorbidities, previous health care resource use, number of dispensed prescriptions, vaccination status and number of previous PCR tests), markers of severity of illness (community, hospital or intensive care within 14 days of index RT-PCR positive result) and PIMS-TS. In order to examine CYP admitted due to SARS-CoV-2 (rather than those with incidental SARS-CoV-2 infection and another reason for admission), a sensitivity analysis will be performed excluding CYP with index SARS-CoV-2 RT-PCR undertaken 72 hours or less before an elective admissions, day case procedure or undertaken at any time during hospitalisation for trauma or emergency surgery.

For peer review only

We will then explore machine learning approaches to identify patterns of healthcare use over time following SARS-CoV-2 infection. Incorporating a machine learning analysis into this study will enable us to examine healthcare use across the 12 month period in a detailed way, investigating whether there are distinct groups of CYP who use healthcare in different ways over this period (i.e. different trajectories). This might be in the level of healthcare used (e.g. GP appointments, outpatient clinics or hospital admissions) or in when they use them (e.g. one group may have higher "upfront" healthcare use in the early period after SARS-CoV-2 infection while another has prolonged high healthcare throughout the 12 month period). Machine learning will allow us to cluster CYP into such trajectory groups and then explore whether particular characteristics are associated with each trajectory. "

We will categorise CYP into groups based on their trajectories (i.e. patterns of healthcare use). Both total healthcare cost and types of healthcare (secondary care and scheduled primary care) will be considered. This will be done using three approaches: a) latent growth mixture model of aggregated healthcare uses over a month [24], b) Bayesian categorical time series clustering of daily service uses of different types [25], and c) centroid based clustering with dynamic time warping distance of smoothed healthcare use cost [26]. By modelling this time series of healthcare use, we will group patients into clusters with similar patterns, e.g., one cluster may correspond to CYP who use general practices on a frequent basis but are not admitted to hospital while another cluster may belong to CYP who do not use general practices but attend outpatient clinics regularly.

After identifying CYP clusters, characteristics (including demographics, comorbidities and previous healthcare use) will be examined to identify any factors which may associated with higher healthcare needs post SARS-CoV-2. These analyses will be stratified by hospitalisation (i.e. hospital admission within 14 days of index RT-PCR positive result) or community care and by diagnosis of PIMS-TS. A sensitivity analysis excluding CYP with presumed incidental SARS-CoV-2 will be carried out as detailed above.

Sensitivity analysis

It is likely that the majority of healthcare costs will be experienced within the first three months of SARS-CoV-2 infection [9]. Following on from Objectives 2 and 3, we will extend the end date of the cohort to three months before the date of data extraction, and examine

healthcare use in the three months following infection with SARS-CoV-2. This will enable us to examine healthcare with later Delta (B.1.617.2) and Omicron (B.1.1.529) variants.

Anticipated limitations

Whilst this protocol has been carefully developed there are anticipated limitations due to constraints of the data. Given the study period, it will also only be possible to examine the annual healthcare costs following infections with wildtype or Alpha (B.1.1.7) SARS-CoV-2 variant infections which may not be the same as after Delta (B.1.617.2) or Omicron (B.1.1.529) variant infections. Data on SARS-CoV-2 viral variant is not consistently available for all CYP in this study. As such, time of RT-PCR testing will be used as a proxy for circulating viral variant at that time. The datasets included do not contain information on SARS-CoV-2 lateral flow testing results which could result in misattribution of SARS-CoV-2 status in patients if RT-PCR testing was not performed. This is likely to particularly affect the later months of the study period where the highly transmissible Omicron variant was widespread and government advice no longer advocated RT-PCR following a positive lateral flow test in some situations [27]. In addition, the study will presume that healthcare services were available for anyone who needed them, but this may have been affected by local healthcare policies and patient health-seeking behaviour at different points during the pandemic. Whilst this study will investigate healthcare use in the 12 months after SARS-CoV-2 infection, there will be other reasons for healthcare contacts in CYP which are not attributable to initial infection which cannot be accounted for in this analysis. This study only examines SARS-CoV-2 infections, not other viral or bacterial infections. It is possible that susceptibility to other infections is not the same in the SARS-CoV-2 RT-PCR positive and negative groups, potentially resulting in more healthcare contacts if one group has more non-SARS-CoV-2 infections over the study period than the other.

Finally, every observational study design has its own limitations. The design of this study relies on CYP registered with a GP which may introduce selection bias against those who are not registered (e.g. in temporary accommodation) as well as the potential for recording biases in individuals coding the healthcare data.

For peer review only

Patient and Public Involvement and Engagement (PPIE)

This proposal was developed together with the Liverpool Generation-R Young Person's Advisory Group (YPAG), a group of engaged CYP aged between 12 and 21 years with lived experience of the SARS-CoV-2 pandemic. A member of the YPAG is also a co-investigator and member of the steering committee, helping ensure the study is delivered appropriately and that decisions about study implementation are guided by meaningful PPIE input. We will undertake two interactive workshops with the YPAG to co-create educational materials for use in schools/science fairs. We will also use these workshops to discuss challenges regarding misinformation about SARS-CoV-2, strategies to correctly share information to young people using social media and the use of routine data in research. The YPAG have named the study – "Studying the Long-term Impact of COVID-19 in Kids (SLICK)" and chosen the logo (*Supplementary Figure F*).

Ethics and Dissemination

This study was approved by the South Central - Oxford C - Health Research Authority Research Ethics Committee, approval reference number 13/SC/0149. This study involves routinely collected anonymised data and as such participant consent was not required.

The EAVE-II dataset was approved by the National Research Ethics Service Committee, South East Scotland 02 (REC number: 12/SS/0201) and the Public Benefit and Privacy Panel for Health and Social Care (reference number: 1920-0279).

EAVE-II was established to provide real time surveillance and research on the SARS-CoV-2 pandemic in Scotland. The study includes the objective of understanding COVID-19 natural history and long term sequalae through studying healthcare utilisation across the primary-secondary-tertiary care interface.

OpenSAFELY is a secure, transparent, open-source software platform for analysis of electronic health records data with all activity publicly logged. The establishment of the OpenSAFELY platform was approved by the Health Research Authority (REC reference 20/LO/0651). The OpenSAFELY research platform adheres to the data protection principles

For peer review only

of the UK Data Protection Act 2018 and the EU General Data Protection Regulation (GDPR) 2016 (for further details please see supplementary information).

The Welsh Con-COV research platform was created to determine demographic, socioeconomic and clinical risk factors for infection and mortality of COVID-19, to measure impact of COVID-19 on healthcare utilisation and long-term health, and to enable the evaluation of natural experiments of policy intervention [28]. The project (SAIL 0911) was approved by the independent Information Governance Review Panel (IGRP). Investigation of the long-term healthcare burden of COVID-19 in children falls under this remit thus Con-COV is approved for use. Approved researchers are also able to access additional information within Con-COV that has been brought to SAIL under the Digital Economy Act (DEA) to Accredited Researchers via the SAIL Databank [29].

Guidelines for the Strengthening the Reporting of Observational Studies in Epidemiology (STROBE) and REporting of studies Conducted using Observational Routinely-collected Data (RECORD) (via the COVID-19 extension) will be followed to report findings from this study. Findings will be presented at international conferences and published in peer-reviewed journals. Reports will also be prepared for policy makers. All analysis code will be made available through a public GitHub repository. In addition, a methods guide to producing harmonised metrics of paediatric healthcare costs across the three nations will be developed with associated code. Code lists to map and classify long term health conditions in paediatric populations in routine primary and secondary care datasets will be made available through OpenCodelists (www.opencodelists.org). Meta-data will be made available via the HDR-UK Innovation Gateway.

Funding

This research is part of the Data and Connectivity National Core Study, led by Health Data Research UK in partnership with the Office for National Statistics and funded by UK Research and Innovation (grant ref MC_PC_20058). This work was also supported by The Alan Turing Institute via 'Towards Turing 2.0' EPSRC Grant Funding. The grant period spans 01/11/21 to 30/09/22.

For peer review only

Acknowledgements

The cohorts included in this work have been funded separately as follows:

ISARIC / CO-CIN is supported by grants from the National Institute for Health Research (award CO-CIN-01) and the Medical Research Council (grant MC_PC_19059) and by the National Institute for Health Research Health Protection Research Unit (NIHR HPRU) in Emerging and Zoonotic Infections at University of Liverpool in partnership with Public Health England (PHE), in collaboration with Liverpool School of Tropical Medicine and the University of Oxford (NIHR award 200907), Wellcome Trust and Department for International Development (215091/Z/18/Z), and the Bill and Melinda Gates Foundation (OPP1209135).

EAVE II is funded by the Medical Research Council [MR/R008345/1] and supported by the Scottish Government. This work is supported by BREATHE—The Health Data Research Hub for Respiratory Health [MC_PC_19004]. BREATHE is funded through 10 the UK Research and Innovation Industrial Strategy Challenge Fund and delivered through Health Data Research UK.

OpenSAFELY is jointly funded by UKRI [COV0076;MR/V015737/1] NIHR and Asthma UK-BLF and the Longitudinal Health and Wellbeing strand of the National Core Studies programme. The OpenSAFELY data science platform is funded by the Wellcome Trust. BG's work on better use of data in healthcare more broadly is currently funded in part by: the Wellcome Trust, NIHR Oxford Biomedical Research Centre, NIHR Applied Research Collaboration Oxford and Thames Valley, the Mohn-Westlake Foundation; all DataLab staff are supported by BG's grants on this work.

SAIL Databank is funded by Health Care Research Wales and the analysis of this work was also funded by Health Care Research Wales through the Centre for Population Health and through Health Data Research Wales/N.Ireland, which receives its funding from HDR UK Ltd (HDR-9006) funded by the UK Medical Research Council, Engineering and Physical Sciences Research Council, Economic and Social Research Council, Department of Health and Social Care (England), Chief Scientist Office of the Scottish Government Health and Social Care Directorates, Health and Social Care Research and Development Division (Welsh Government), Public Health Agency (Northern Ireland), British Heart Foundation

For peer review only

(BHF) and the Wellcome Trust. This work was supported by the Con-COV team funded by the Medical Research Council (grant number: MR/V028367/1).

Funders had no role in the study design, collection, analysis, and interpretation of data; in the writing of the report; and in the decision, submit the article for publication.

Author's contributions

OVS, NIL, EMH, PSH, MGS, SB, BG and ABD were responsible for conception of this project. OVS, LAT , AJW, MJS, JF, BG, SB and ABD will be responsible for data curation. OVS, NIL, EMH, LAT, AJW, MJS, JF, SS and ABD will be undertaking the analysis for this protocol. OVS, NIL, EMH, JKB, MGS, BG, SB, AS and ABD were responsible for securing funding for this project or its constituent cohorts. OVS, NIL, EMH, LAT, AJW, MJS, LP, JF, PSH, SS, JP, JSA, FFS, SVK, CRS, MGS, SB and ABD designed the analysis plan. OVS and ABD are providing administrative support to this project. LAT, AJW, MS, JP, JA, FFS, JKB, AA, RL, MGS, BG, SB, AS and ABD are providing resources to this project. EMH, LAT, AJW, MJS, SS and BG are providing software for this project. MGS, AS and ABD are providing supervision. EMH, LAT, AJW, MJS, JF, TCW and SS will be responsible for data validation. OVS, EMH, AJW, MJS and SS are responsible for data visualisation. OVS, NIL, EMH, LAT, AJW, MJS, LP, PSH, SS and ABD wrote the original draft of this protocol and all authors were involved in the review and editing of this manuscript.

Competing Interests

OVS reports an institutional payment from HDR-UK/Alan Turing for work on this study. **LAT** reports institutional contracts with UKRI, NIHR, MRC, institutional consulting fees from Bayer, support to attend MHRA meetings and unpaid membership of two non-industry funded trial advisory committees. **MS** reports an institutional payment from HDR-UK/Alan Turing for work on this study. **CRS** reports institutional grants from MBIE, HRC and MRC. **SVK** reports funding from NRS, MRC and the Scottish Government Chief Scientist Office. He was co-chair of the Scottish Government's Expert Reference Group on Ethnicity and COVID-19 and a member of the UK Scientific Advisory Group on Emergencies subgroup on

For peer review only

ethnicity. **MGS** reports grants from NIHR, MRC and Health Protection Research Unit in Emerging & Zoonotic Infections, University of Liverpool. He also reports a role as Independent external and non-remunerated member of Pfizer's External Data Monitoring Committee for their mRNA vaccine program. He is Chair of Infectious Disease Scientific Advisory Board for Integrum Scientific LLC, Greensboro, NC, USA and director of MedEx Solutions Ltd. He reports minority stock ownership for Integrum Scientific LLC, Greensboro, NC, USA and majority stock ownership for MedEx Solutions Ltd. He also reports a gift from Chiesi Farmaceutici SPA to his institution of a clinical trial investigational medicinal product without encumbrance and distribution of same to trial sites. He is also a non-remunerated independent member of HMG UK Scientific Advisory Group for Emergencies (SAGE, COVID-19 Response) and HMG UK New Emerging Respiratory Virus Threats Advisory Group (NERVTAG). **SB** has received an institutional payment from HDR-UK/Alan Turing funding UOE Ref: 11563729 for work on this study. She also reports institutional payments from MRC, Welsh Government and NIHR. She is a member of the Population and Systems Medicine MRC board. **AS** reports an institutional payment from HDR-UK/Alan Turing and research grants for EAVE II and BREATHE Hub. He also reports non-remunerated positions on AstraZeneca's Thrombotic Thrombocytopenic Taskforce and Scottish and UK Government Advisory Committees. RAL is a member of the Welsh Government COVID-19 Technical Advisory Group. **BG** has received research funding from HDRUK, the Laura and John Arnold Foundation, the Wellcome Trust, the NIHR Oxford Biomedical Research Centre, the NHS National Institute for Health Research School of Primary Care Research, the Mohn-Westlake Foundation, the Good Thinking Foundation, the Health Foundation, and the World Health Organisation; he also receives personal income from speaking and writing for lay audiences on the misuse of science.

AJW, NIL, EMH, LP, JF, PSH, SS, AA, TCW, JP, JSA, FFS, JKB and **ABD** report no competing interests.

For peer review only

References

1. Docherty AB, Harrison EM, Green CA, et al. Features of 20 133 UK patients in hospital with covid-19 using the ISARIC WHO Clinical Characterisation Protocol: prospective observational cohort study. *BMJ*; 369. Epub ahead of print 22 May 2020. DOI: 10.1136/bmj.m1985.

2. Swann OV, Holden KA, Turtle L, et al. Clinical characteristics of children and young people admitted to hospital with covid-19 in United Kingdom: prospective multicentre observational cohort study. *BMJ*; 370. Epub ahead of print 27 August 2020. DOI: 10.1136/bmj.m3249.

3. Birrel P, Blake J, van Leeuwen E, et al. Report on Nowcasting and Forecasting - 9th December 2021. *MRC Biostatistics Unit*, https://www.mrc-bsu.cam.ac.uk/now-casting/report-on-nowcasting-and-forecasting-9th-december-2021/ (accessed 9 February 2022).

4. Whittaker E, Bamford A, Kenny J, et al. Clinical Characteristics of 58 Children With a Pediatric Inflammatory Multisystem Syndrome Temporally Associated With SARS-CoV-2. *JAMA*. Epub ahead of print 8 June 2020. DOI: 10.1001/jama.2020.10369.

5. Payne AB, Gilani Z, Godfred-Cato S, et al. Incidence of Multisystem Inflammatory Syndrome in Children Among US Persons Infected With SARS-CoV-2. *JAMA Netw Open* 2021; 4: e2116420.

6. Behnood SA, Shafran R, Bennett S, et al. *Persistent Symptoms Following SARS-CoV-2 Infection Among Children and Young People: A Meta-Analysis of Controlled and Uncontrolled Studies*. SSRN Scholarly Paper ID 3940260, Rochester, NY: Social Science Research Network. Epub ahead of print 12 October 2021. DOI: 10.2139/ssrn.3940260.

7. Al-Aly Z, Xie Y, Bowe B. High-dimensional characterization of post-acute sequelae of COVID-19. *Nature* 2021; 594: 259–264.

8. Koumpias AM, Schwartzman D, Fleming O. Long-haul COVID: healthcare utilization and medical expenditures 6 months post-diagnosis. *BMC Health Serv Res* 2022; 22: 1010.

9. Magnusson K, Skyrud KD, Suren P, et al. Healthcare use in 700 000 children and adolescents for six months after covid-19: before and after register based cohort study. *BMJ* 2022; 376: e066809.

10. Zoabi Y, Deri-Rozov S, Shomron N. Machine learning-based prediction of COVID-19 diagnosis based on symptoms. *Npj Digit Med* 2021; 4: 1–5.

11. Office for National Statistics. Coronavirus (COVID-19) Infection Survey technical article: waves and lags of COVID-19 in England, June 2021, https://www.ons.gov.uk/peoplepopulationandcommunity/healthandsocialcare/conditionsanddiseases/articles/coronaviruscovid19infectionsurveytechnicalarticle/wavesandlagsofcovid19inenglandjune2021 (2021, accessed 17 December 2021).

12. Kontopantelis E, Stevens RJ, Helms PJ, et al. Spatial distribution of clinical computer systems in primary care in England in 2016 and implications for primary care electronic medical record databases: a cross-sectional population study. *BMJ Open* 2018; 8: e020738.

13. Surveillance definitions for COVID-19. *European Centre for Disease Prevention and Control*, https://www.ecdc.europa.eu/en/covid-19/surveillance/surveillance-definitions (accessed 17 December 2021).

14. Williamson EJ, Walker AJ, Bhaskaran K, et al. OpenSAFELY: factors associated with COVID-19 death in 17 million patients. *Nature* 2020; 584: 430–436.

15. Lyons RA, Jones KH, John G, et al. The SAIL databank: linking multiple health and social care datasets. *BMC Med Inform Decis Mak* 2009; 9: 3.

16. Simpson CR, Robertson C, Vasileiou E, et al. Early Pandemic Evaluation and Enhanced Surveillance of COVID-19 (EAVE II): protocol for an observational study using linked Scottish national data. *BMJ Open* 2020; 10: e039097.

17. Zhu Y, Matsuyama Y, Ohashi Y, et al. When to conduct probabilistic linkage vs. deterministic linkage? A simulation study. *J Biomed Inform* 2015; 56: 80–86.

18. Fraser LK, Murtagh FEM, Sheldon T, et al. Health of mothers of children with a life-limiting condition: a protocol for comparative cohort study using the Clinical Practice Research Datalink. *BMJ Open* 2020; 10: e034024.

19. Ward JL, Harwood R, Smith C, et al. Risk factors for PICU admission and death among children and young people hospitalized with COVID-19 and PIMS-TS in England during the first pandemic year. *Nat Med* 2021; 1–8.

For peer review only

20. Adeloye D, Katikireddi SV, Woolford L, et al. Uptake, effectiveness and safety of COVID-19 vaccines in children and young people in Scotland: protocol for early pandemic evaluation and enhanced surveillance of COVID-19 (EAVE II). *J Glob Health*.

21. Curtis LA, Burns A. *Unit Costs of Health & Social Care 2020*. PSSRU, University of Kent, https://www.pssru.ac.uk/project-pages/unit-costs/unit-costs-2020/ (2020, accessed 9 December 2021).

22. Swann OV, Pollock L, Holden KA, et al. Comparison of children and young people admitted with SARS-CoV-2 across the UK in the first and second pandemic waves: prospective multicentre observational cohort study. 2021; 2021.09.14.21263567.

23. Green MA, García-Fiñana M, Barr B, et al. Evaluating social and spatial inequalities of large scale rapid lateral flow SARS-CoV-2 antigen testing in COVID-19 management: An observational study of Liverpool, UK (November 2020 to January 2021). *Lancet Reg Health - Eur* 2021; 6: 100107.

24. Jung T, Wickrama K a. S. An Introduction to Latent Class Growth Analysis and Growth Mixture Modeling. *Soc Personal Psychol Compass* 2008; 2: 302–317.

25. Frühwirth-Schnatter S, Pamminger C. Model-based clustering of categorical time series. *Bayesian Anal* 2010; 5: 345–368.

26. Franses PH, Wiemann T. Intertemporal Similarity of Economic Time Series: An Application of Dynamic Time Warping. *Comput Econ* 2020; 56: 59–75.

27. People with a positive lateral flow test no longer required to take confirmatory PCR test. *GOV.UK*, https://www.gov.uk/government/news/people-with-a-positive-lateral-flow-test-no-longer-required-to-take-confirmatory-pcr-test (accessed 18 January 2022).

28. Lyons J, Akbari A, Torabi F, et al. Understanding and responding to COVID-19 in Wales: protocol for a privacy-protecting data platform for enhanced epidemiology and evaluation of interventions. *BMJ Open* 2020; 10: e043010.

29. Jones KH, Ford DV, Jones C, et al. A case study of the Secure Anonymous Information Linkage (SAIL) Gateway: a privacy-protecting remote access system for health-related research and evaluation. *J Biomed Inform* 2014; 50: 196–204.

For peer review only

Figure 1. Graphical illustrations of potential study scenarios with test negative controls.

Example A: Positive SARS-CoV-2 RT-PCR case. **Individual A** is followed-up from 14 days after SARS-CoV-2 infection for 12 months. **Examples B-D: Test negative controls. Individual B** is matched to an individual with SARS-CoV-2 infection and followed-up from 14 days after matching for 12 months. **Individual C** is matched to an individual with SARS-CoV-2 infection and followed-up from 14 days after matching until they are first recorded with SARS-CoV-2 infection themselves during the RT-PCR testing period of interest. At this point they are censored from further follow-up as a test negative comparator and followed-up as an exposed case from 14 days after infection for 12 months with appropriate matches for the date of positive RT-PCR. **Individual D** is matched to an individual with SARS-CoV-2 infection and followed-up from 14 days after matching until they are first recorded with SARS-CoV-2 infection themselves. As this occurs after the RT-PCR testing period of interest, they are censored from further follow-up as an unexposed comparator.

For peer review only

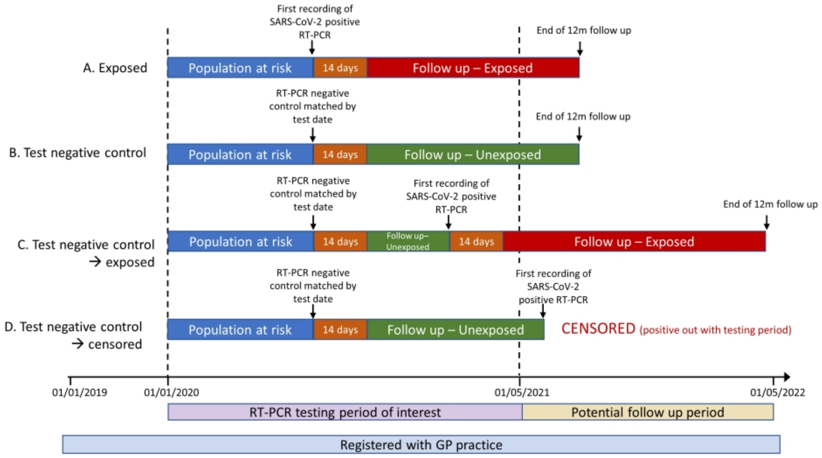

Figure 1. Graphical illustrations of potential study scenarios with test negative controls.

Example A: Positive SARS-CoV-2 RT-PCR case.
Individual A is followed-up from 14 days after SARS-CoV-2 infection for 12 months.

Examples B-D: Test negative controls.
Individual B is matched to an individual with SARS-CoV-2 infection and followed-up from 14 days after matching for 12 months.
Individual C is matched to an individual with SARS-CoV-2 infection and followed-up from 14 days after matching until they are first recorded with SARS-CoV-2 infection themselves during the RT-PCR testing period of interest. At this point they are censored from further follow-up as a test negative comparator and followed-up as an exposed case from 14 days after infection for 12 months with appropriate matches for the date of positive RT-PCR.
Individual D is matched to an individual with SARS-CoV-2 infection and followed-up from 14 days after matching until they are first recorded with SARS-CoV-2 infection themselves. As this occurs after the RT-PCR testing period of interest, they are censored from further follow-up as an unexposed comparator.

338x190mm (300 x 300 DPI)

Studying the Long-term Impact of COVID-19 in Kids (SLICK). Healthcare use and costs in children and young people following community-acquired SARS-CoV-2 infection: protocol for an observational study using linked primary and secondary routinely collected healthcare data from England, Scotland and Wales.

Supplementary Information

Supplementary Table 1 – Datasets available and Trusted Research Environments

Country	Trusted Research Environment	Datasets and linkages
Scotland	Scottish National Safe Haven	**SMR00** – Outpatient appointments and attendances<br>**SMR01** – General acute inpatient and day case<br>**SMR04** – Mental health inpatient and day case<br>**SMR06** – Scottish cancer registry<br>**COVID Tests** – Laboratory SARS-CoV-2 tests<br>**Prescribing Information System** – Community prescriptions<br>**Accident and Emergency Datamart**<br>**GP out of hours**<br>**Scottish Ambulance Service**<br>**NHS24 calls**<br>**NRS Deaths**<br>**NRS Infant deaths**<br>**EAVE II** – Scheduled and unscheduled primary care<br>**ISARIC4C/CO-CIN**<br>**COGUK** – SARS-CoV-2 variant<br>**TVMT** – SARS-Cov-2 vaccination data
England	OpenSAFELY	**TPP** - Primary Care<br>**SGSS COVID testing data**<br>**ONS death certificates** – available from 2019-02-01<br>**SUS APCS** (inpatient hospital) – available from 2016-04-01<br>**SUS OPA** (outpatient hospital) – available from 2019-04-01<br>**SUS ECDS** (emergency care) – available from 2017-10-01<br>**ISARIC4C/CO-CIN**
Wales	SAIL	**ConCOV** - Wales Multimorbidity Cohort (WMC) - COVID-19<br>**WLGP** – Primary care<br>**PEDW** – Secondary care (inpatient & day case)<br>**ADDE** – ONS mortality data<br>**CCDS** – Critical care<br>**CDDS** – Consolidate deaths from COVID-19

For peer review only

		**CENW** – Census 2011
		**CTTP** – COVID-19 test, trace and protect
		**CVLF** – COVID-19 lateral flow tests
		**CVSP** – COVID-19 shielded people
		**CVVD** – COVID-19 vaccines
		**EDDD** – Emergency department (daily)
		**EDDS** – Emergency department
		**ICCD** – intensive care national audit (COVID only admissions)
		**ICNC** – intensive care national audit
		**MIDS** – Maternity initial screening and birth
		**NCCH** – National community child health (maternity, childbirth, etc)
		**NHSO** – NHS 111, out of hours
		**OPDW** – Outpatients
		**OPRD** – Outpatient referrals
		**PATD** – COVID-19 lab tests
		**RTTD** – Referral to treatment times
		**WASD** – Welsh ambulance service
		**WCSU** – Welsh cancer incidence surveillance unit
		**WDDS** – Welsh prescription dispensing
		**WDSD** – Individuals registered with GP, addresses/household information
		**CRCS** – Children in care or receiving support register
		**CYFI** – Cystic Fibrosis register
		**BREC** – Register of all children in Wales with type 1 diabetes
		**ISARIC4C/CO-CIN**

***Abbreviations: SMR**: Scottish Morbidity Record, **NRS**: National Records of Scotland **EAVE-II:** Early Pandemic Evaluation and Enhanced Surveillance of COVID-19, **ISARIC/CO-CIN**: International Severe Acute Respiratory and emerging Infection Consortium / COVID-19 Clinical Information Network, **COGUK:** COVID-19 Genomics UK Consortium, **TMVT**:Turas Vaccination Management Tool, **NHS**: National Health Service, **TPP:** The Phoenix Partnership (GP group), **SGSS**: Second Generation Surveillance System, **ONS**: Office for National Statistics, **SUS**: Secondary Use Services, **APCS**: Admitted patient care statistics, **OPA**: Outpatient attendances, **ECDS**: Emergency care datasets, **SAIL:** Secure Anonymised Information Linkage, **WLGP**: Welsh Longitudinal General Practice, **PEDW**: Patient Episode Database for Wales, **ADDE**: Annual District Death Extract, **CCDS:** Critical Care Data Source, **CDDS:** COVID-19 Consolidated Deaths, **CENW:** Office of National Statistics Census, **CTTP:** COVID-19 Test, Trace & Protect, **CVLF:** COVID-19 Lateral Flow, **CVSP:** COVID-19 Shielded People, **CVVD:** COVID-19 Vaccine Data, **EDDD:** Emergency Department Dataset Daily, **EDDS:** Emergency Department Dataset, **ICCD:** Intensive Care National Audit & Research Centre (ICNARC) - COVID only admissions, **ICNC:** Intensive Care National Audit & Research Centre (ICNARC), **MIDS:** Maternity Indicators Dataset, **NCCH:** National Community Child Health, **NHSO:** NHS 111 Call data, **OPDW:** Outpatient Dataset for Wales, **OPRD:** Outpatient Referral Dataset, **PATD:** Pathology Data (COVID-19 daily), **RTTD:** Referral to Treatment Times Dataset, **WASD:** Welsh Ambulance Service Dataset, **WCSU:** Welsh Cancer Incidence Surveillance Unit, **WDDS:** Welsh Dispensing Dataset, **WDSD:** Welsh Demographic Service Dataset.*

For peer review only

Supplementary Figure A – Dataflow diagram for Wales

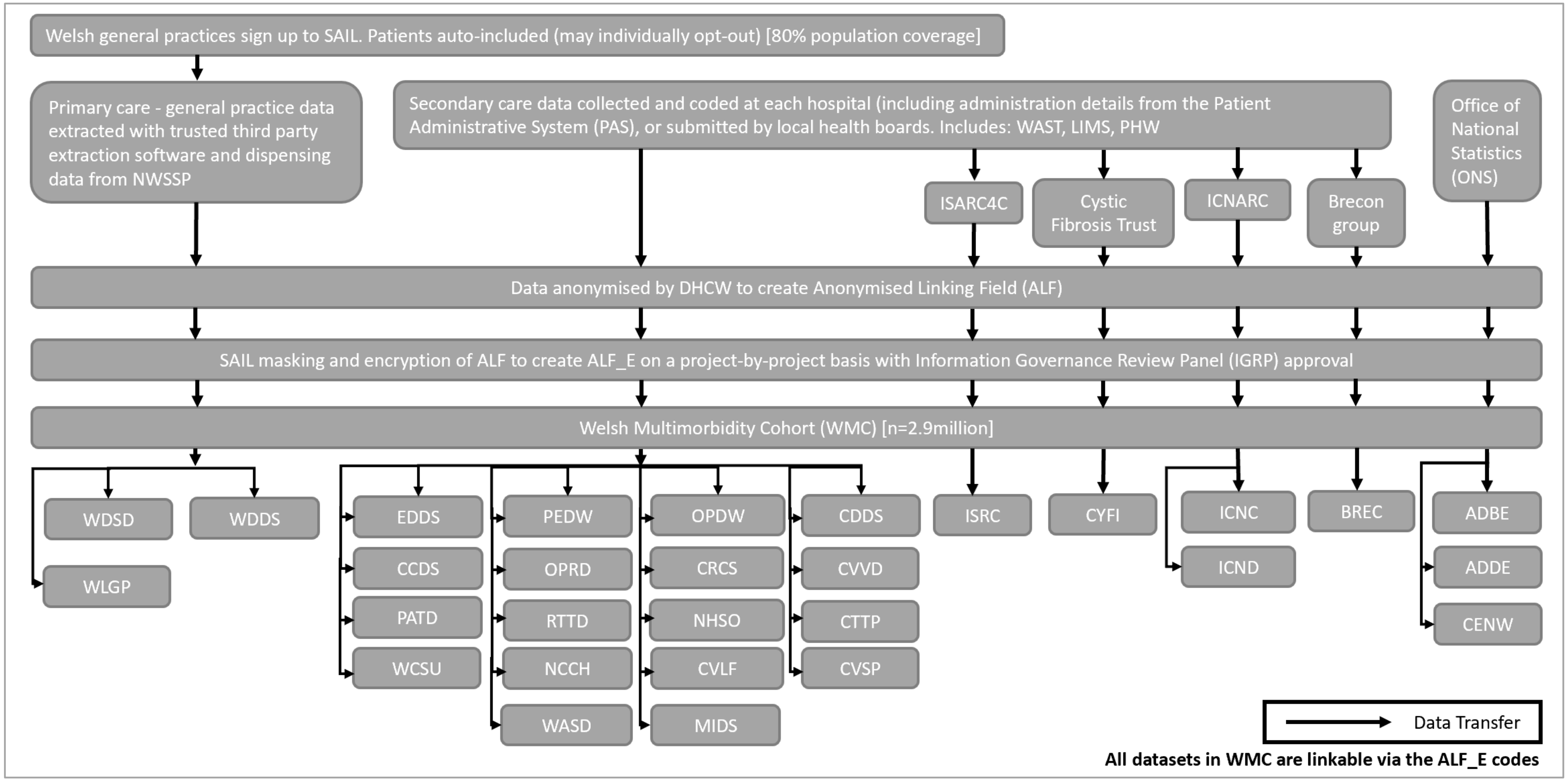

***Abbreviations: SAIL:** Secure Anonymised Information Linkage, **WLGP**: Welsh Longitudinal General Practice, **PEDW**: Patient Episode Database for Wales, **ADDE**: Annual District Death Extract, **BREC:** Brecon Cohort (Children with type 1 diabetes register), **CCDS:** Critical Care Data Source, **CDDS:** COVID-19 Consolidated Deaths, **CENW:** Office of National Statistics Census, **CRCS:** Children Receiving Care & Support Services, **CTTP:** COVID-19 Test, Trace & Protect, **CVLF:** COVID-19 Lateral Flow, **CVSP:** COVID-19 Shielded People, **CVVD:** COVID-19 Vaccine Data, **CYFI:** Cystic Fibrosis Register, **EDDS:** Emergency Department Dataset, **ICCD:** Intensive Care National Audit & Research Centre (ICNARC) - COVID only admissions, **ICNC:** Intensive Care National Audit & Research Centre (ICNARC), **ISRC:** International Severe Acute Respiratory & Emerging Infection Consortium, **ISARIC4C:** International Severe Acute Respiratory & Emerging Infection Consortium (Coronavirus Clinical Characterisation Consortium), **MIDS:** Maternity Indicators Dataset, **NCCH:** National Community Child Health, **NHSO:** NHS 111 Call data, **OPDW:** Outpatient Dataset for Wales, **OPRD:** Outpatient Referral Dataset, **PATD:** Pathology Data (COVID-19 daily), **RTTD:** Referral to Treatment Times Dataset, **WASD:** Welsh Ambulance Service Dataset, **WCSU:** Welsh Cancer Incidence Surveillance Unit, **WDDS:** Welsh Dispensing Dataset, **WDSD:** Welsh Demographic Service Dataset.*

Supplementary Figure B – Dataflow diagram for Scotland

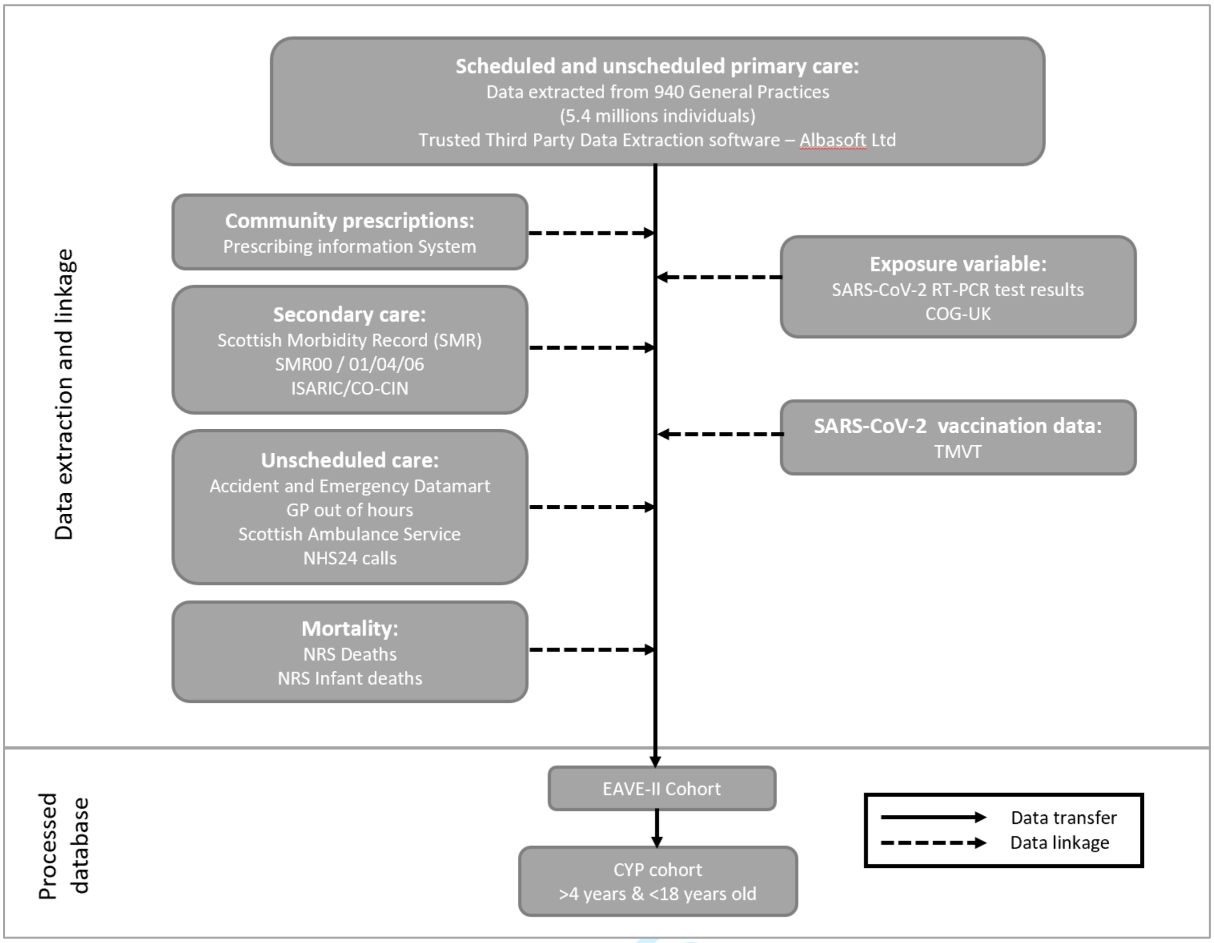

***Abbreviations: ISARIC/CO-CIN**: International Severe Acute Respiratory and emerging Infection Consortium / COVID-19 Clinical Information Network, **GP**: general practice; **NHS**: National Health Service, **NRS**: National Records of Scotland; **RT-PCR**: reverse transcription polymerase chain reaction, **TMVT**: Turas Vaccination Management Tool; **COG-UK:** Centre of Genomics United Kingdom, **CYP**: children and young people*

For peer review only - http://bmjopen.bmj.com/site/about/guidelines.xhtml

Supplementary Figure C – Dataflow diagram for England

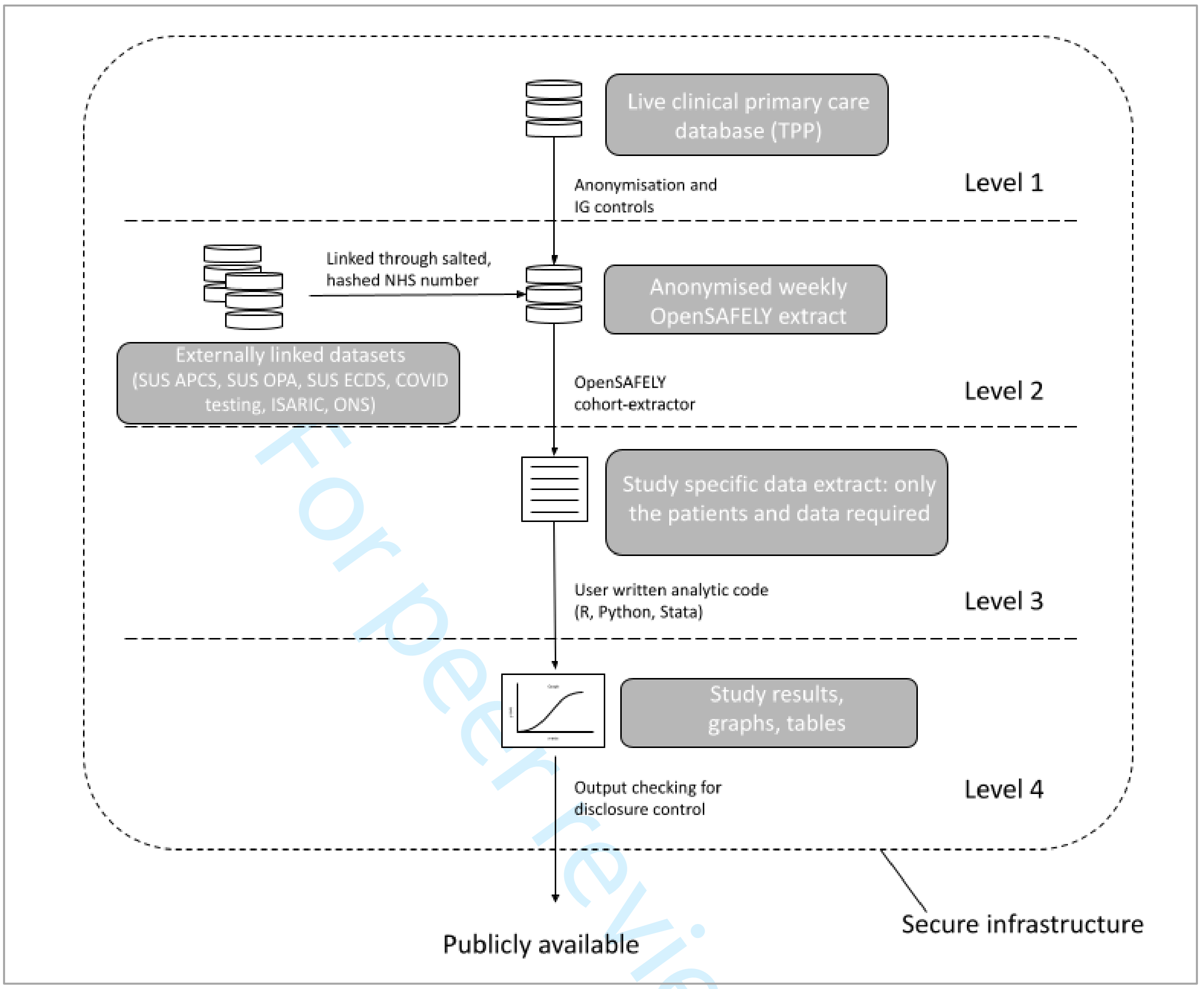

__Abbreviations: TPP:__ The Phoenix Partnership (GP group), __SGSS__: Second Generation Surveillance System, __ONS__: Office for National Statistics, __SUS__: Secondary Use Services, __APCS__: Admitted patient care statistics, __OPA__: Outpatient attendances, __ECDS__: Emergency care datasets, __ONS__: Office for National Statistics.

For peer review only

Supplementary Figure D– Directed acyclic graph factors associated with SARS-CoV-2 RT-PCR testing and healthcare use

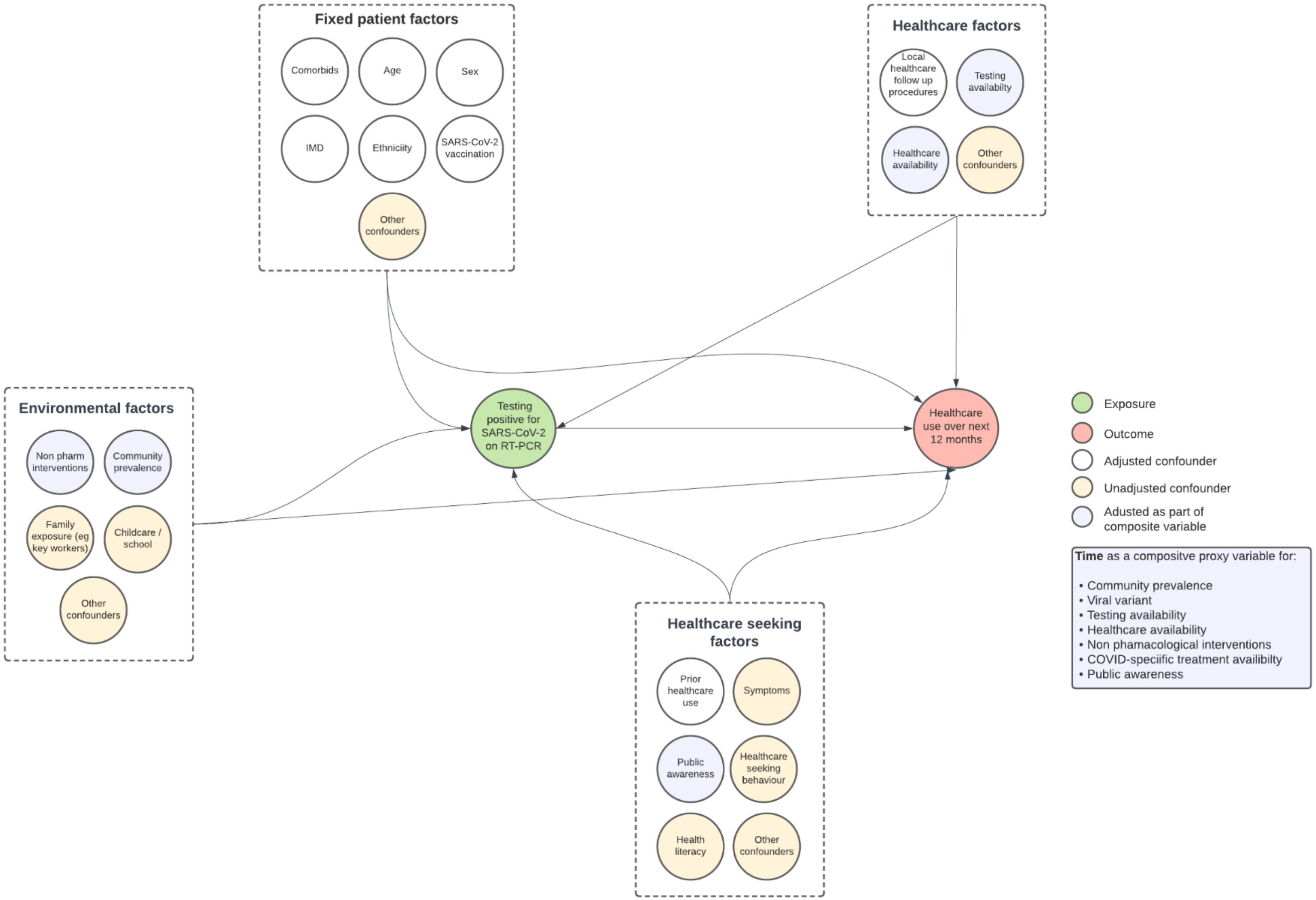

Supplementary Figure E. Graphical illustration of potential study scenarios with population controls (Objective 2 sensitivity analysis)

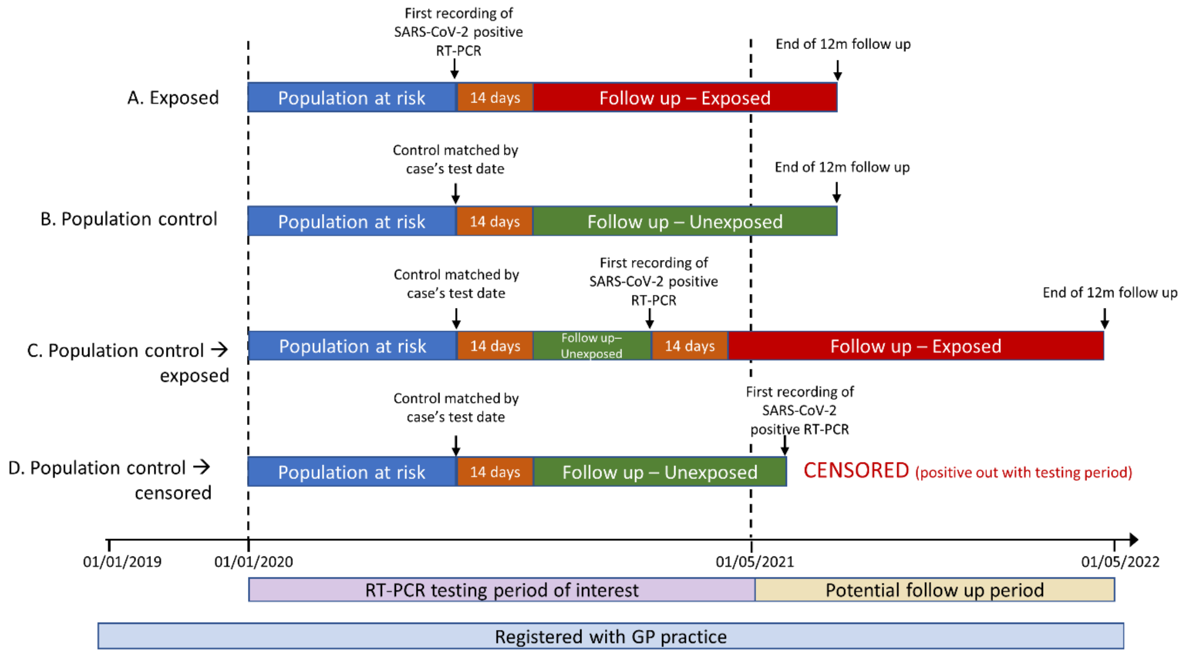

Example A: Positive SARS-CoV-2 RT-PCR case. Individual A is followed-up from 14 days after SARS-CoV-2 infection for 12 months. **Examples B-D: Population controls. Individual B** is matched to an individual with SARS-CoV-2 infection and followed-up from 14 days after matching for 12 months. **Individual C** is matched to an individual with SARS-CoV-2 infection and followed-up from 14 days after matching until they are first recorded with SARS-CoV-2 infection themselves during the RT-PCR testing period of interest. At this point they are censored from further follow-up as a test negative comparator and followed-up as an exposed case from 14 days after infection for 12 months with appropriate matches for the date of positive RT-PCR. **Individual D** is matched to an individual with SARS-CoV-2 infection and followed-up from 14 days after matching until they are first recorded with SARS-CoV-2 infection themselves. As this occurs after the RT-PCR testing period of interest, they are censored from further follow-up as an unexposed comparator.

For peer review only - http://bmjopen.bmj.com/site/about/guidelines.xhtml

Supplementary Figure F – Logo for SLICK study

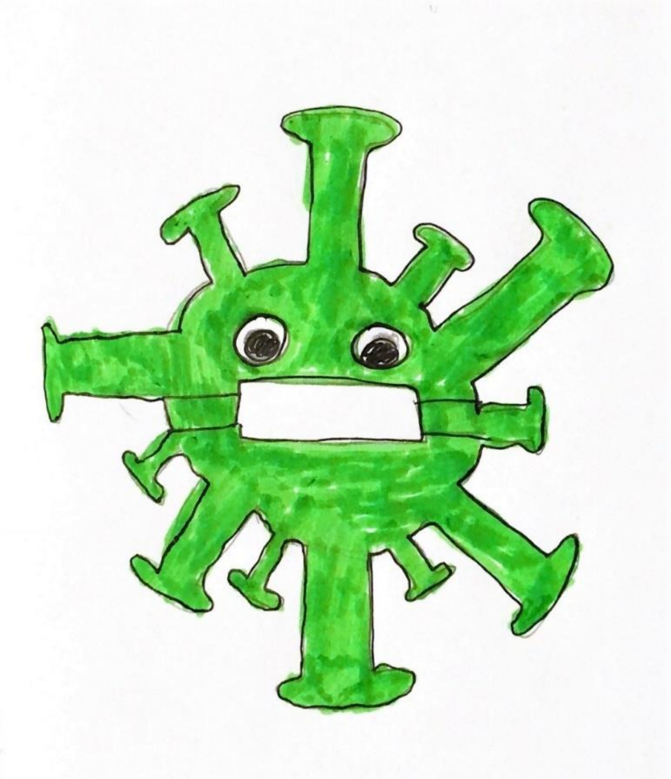

Designed by Georgia Langley, aged 11 (consent obtained from parent to publish child's name)

OpenSAFELY Information governance and ethical approval – further information

NHS England is the data controller for OpenSAFELY-TPP; TPP is the data processor; all study authors using OpenSAFELY have the approval of NHS England. This implementation of OpenSAFELY is hosted within the TPP environment which is accredited to the ISO 27001 information security standard and is NHS IG Toolkit compliant. [1]

Patient data has been pseudonymised for analysis and linkage using industry standard cryptographic hashing techniques; all pseudonymised datasets transmitted for linkage onto OpenSAFELY are encrypted; access to the platform is via a virtual private network (VPN) connection, restricted to a small group of researchers; the researchers hold contracts with NHS England and only access the platform to initiate database queries and statistical models; all database activity is logged; only aggregate statistical outputs leave the platform environment following best practice for anonymisation of results such as statistical disclosure control for low cell counts.[2]

The OpenSAFELY research platform adheres to the obligations of the UK General Data Protection Regulation (GDPR) and the Data Protection Act 2018. In March 2020, the Secretary of State for Health and Social Care used powers under the UK Health Service (Control of Patient Information) Regulations 2002 (COPI) to require organisations to process confidential patient information for the purposes of protecting public health, providing healthcare services to the public and monitoring and managing the COVID-19 outbreak and incidents of exposure; this sets aside the requirement for patient consent.[3] This was extended in July 2022 for the NHS England OpenSAFELY COVID-19 research platform.[4] In

For peer review only - http://bmjopen.bmj.com/site/about/guidelines.xhtml

some cases of data sharing, the common law duty of confidence is met using, for example, patient consent or support from the Health Research Authority Confidentiality Advisory Group.[5]

Taken together, these provide the legal bases to link patient datasets on the OpenSAFELY platform. GP practices, from which the primary care data are obtained, are required to share relevant health information to support the public health response to the pandemic, and have been informed of the OpenSAFELY analytics platform.

Supplementary references:

1. Data Security and Protection Toolkit - NHS Digital. NHS Digital. https://digital.nhs.uk/data-and-information/looking-after-information/data-security-and-information-governance/data-security-and-protection-toolkit (accessed 30 Apr 2020).

2. ISB1523: Anonymisation Standard for Publishing Health and Social Care Data - NHS Digital. NHS Digital. https://digital.nhs.uk/data-and-information/information-standards/information-standards-and-data-collections-including-extractions/publications-and-notifications/standards-and-collections/isb1523-anonymisation-standard-for-publishing-health-and-social-care-data (accessed 30 Apr 2020).

3. Secretary of State for Health and Social Care - UK Government. Coronavirus (COVID-19): notification to organisations to share information. 2020. https://web.archive.org/web/20200421171727/https://www.gov.uk/government/publications/coronavirus-covid-19-notification-of-data-controllers-to-share-information

4. Secretary of State for Health and Social Care - UK Government. Coronavirus (COVID-19): notification to organisations to share information. 2022. https://www.gov.uk/government/publications/coronavirus-covid-19-notification-to-organisations-to-share-information/coronavirus-covid-19-notice-under-regulation-34-of-the-health-service-control-of-patient-information-regulations-2002

5. Confidentiality Advisory Group. Health Research Authority. https://www.hra.nhs.uk/about-us/committees-and-services/confidentiality-advisory-group/

For peer review only

For peer review only - http://bmjopen.bmj.com/site/about/guidelines.xhtml
